# An essential bifunctional enzyme in *Mycobacterium tuberculosis* for itaconate dissimilation and leucine catabolism

**DOI:** 10.1073/pnas.1906606116

**Published:** 2019-07-18

**Authors:** Hua Wang, Alexander A. Fedorov, Elena V. Fedorov, Debbie M. Hunt, Angela Rodgers, Holly L. Douglas, Acely Garza-Garcia, Jeffrey B. Bonanno, Steven C. Almo, Luiz Pedro Sório de Carvalho

**Affiliations:** ^a^Mycobacterial Metabolism and Antibiotic Research Laboratory, The Francis Crick Institute, London NW1 1AT, United Kingdom;; ^b^Department of Biochemistry, Albert Einstein College of Medicine, Bronx, NY 10461

**Keywords:** enzyme function, carbon–carbon bond lyase, *Mycobacterium tuberculosis*, itaconate catabolism, leucine catabolism

## Abstract

*Mycobacterium tuberculosis* (Mtb) is the etiological agent of tuberculosis. One-fourth of the global population is estimated to be infected with Mtb, accounting for ∼1.3 million deaths in 2017. As part of the immune response to Mtb infection, macrophages produce metabolites with the purpose of inhibiting or killing the bacterial cell. Itaconate is an abundant host metabolite thought to be both an antimicrobial agent and a modulator of the host inflammatory response. However, the exact mode of action of itaconate remains unclear. Here, we show that Mtb has an itaconate dissimilation pathway and that the last enzyme in this pathway, Rv2498c, also participates in l-leucine catabolism. Our results from phylogenetic analysis, in vitro enzymatic assays, X-ray crystallography, and in vivo Mtb experiments, identified Mtb Rv2498c as a bifunctional β-hydroxyacyl-CoA lyase and that deletion of the *rv2498c* gene from the Mtb genome resulted in attenuation in a mouse infection model. Altogether, this report describes an itaconate resistance mechanism in Mtb and an l-leucine catabolic pathway that proceeds via an unprecedented (*R*)-3-hydroxy-3-methylglutaryl-CoA (HMG-CoA) stereospecific route in nature.

## Significance

Accounting for 1.3 million deaths in 2017, the bacillus *Mycobacterium tuberculosis* (Mtb) primarily resides within human macrophages. Itaconate is suggested to be an antimicrobial metabolite and immunomodulator produced by macrophages during Mtb infection. Here, we show that Mtb is intrinsically resistant to itaconate and Mtb degrades l-leucine via an unprecedented *R*-stereospecific route. Our work reveals Rv2498c as a bifunctional enzyme involved in itaconate dissimilation and l-leucine catabolism in Mtb.

*Mycobacterium tuberculosis* (Mtb) is the etiological agent of tuberculosis (TB). In 2017, an estimated 10 million people developed TB and around 1.3 million people died from the disease; it is thought that up to one-fourth of the global population is infected with Mtb (1, 2). The pathogenicity, physiological resiliency and plasticity of Mtb are notably complex, with humans serving as their main reservoir, highlighting the effect of niche adaptation on pathogen evolution (3, 4). Despite the medical impact of TB, Mtb biology remains largely unexplored, and over half of the enzymes in the proteome lack a defined catalytic activity (5, 6). To date, delineating the in vivo functional roles of hundreds of poorly characterized enzymes has been a significant challenge (7). This difficulty is in part due to the unconventional nutrient assimilation of Mtb and its ability to persist in different metabolic states (8–11). Without an adequate understanding of the fundamental biology underpinning infection and the associated metabolic networks, we continue to generate drug candidates that fail in eradicating the pathogen. Moreover, our knowledge of the exact cellular and molecular mechanisms by which the immune system eradicates Mtb or fails to do so remains incomplete. Thus, a deeper understanding of the Mtb biology and its interaction with the host is pivotal to tackle the TB pandemic.

Itaconate is produced by the mitochondrial enzyme *cis*-aconitate decarboxylase found in both stimulated and unstimulated macrophages. Itaconate has been reported to be both an antimicrobial metabolite and an immunomodulator, but its exact physiologic role has remained enigmatic and is of growing interest (12–17). Mtb elicits an immune response that leads to a supraphysiological concentration (millimolar range) of itaconate in macrophages; itaconate inhibits the bacterial isocitrate lyase, a key enzyme in the glyoxylate shunt that is known to be associated with bacterial pathogenesis (12, 18, 19). A canonical itaconate dissimilation pathway has been described in vitro and ex vivo in mammalian mitochondria and in bacteria such as *Micrococcus* sp., *Salmonella* sp., *Pseudomonas* sp., and *Yersinia* sp. In these bacteria, itaconate dissimilation involves the activation of itaconate with succinyl-CoA to form itaconyl-CoA, catalyzed by a CoA transferase, followed by stereo-specific hydration to form (*S*)-citramalyl-CoA, catalyzed by a hydratase, and subsequently carbon–carbon bond cleavage to form pyruvate and acetyl-CoA (Ac-CoA), catalyzed by a lyase (20–24). Logically, due to the significant chemical structural resemblance of itaconate and CoA-thioester intermediates to TCA cycle metabolites and endogenous CoA thioesters, we inferred that Mtb might possess an itaconate dissimilation pathway, composed of a currently unidentified transferase, hydratase, and lyase. If such pathway exists in Mtb, as an intracellular pathogen, it might take advantage of the high concentration of itaconate present in macrophages and use itaconate as a carbon source (3, 4).

Mtb protein Rv2498c is currently annotated as the β-subunit (CitE) of the heterotrimeric prokaryotic citrate lyase complex involved in TCA cycle cataplerosis (25–27). However, the genes encoding the associated α- and γ-subunits of the citrate lyase complex are absent in the Mtb genome, suggesting a different but related function (25, 28). Bona fide CitE enzymes catalyze the cleavage of (3*S*)-citryl-CoA to Ac-CoA and oxaloacetate (Enzyme Commission [EC] 4.1.3.34), thus Rv2498c is most probably also a short-chain acyl-CoA lyase but of unknown substrate specificity and physiologic role.

Combining phylogenetic analysis, in vitro enzymatic assays, in vivo Mtb experiments, and X-ray crystallography, we identified Mtb Rv2498c as an essential stereospecific bifunctional β-hydroxyacyl-CoA lyase (β-HAC lyase) that carries out the last step of itaconate dissimilation pathway and confers resistance to itaconate. Overall, these findings offer insights into the biology and physiological plasticity of Mtb.

## Results

### Phylogenetic Analysis Identifies Two Putative Mtb Lyases.

*Rv2498c* is reported to encode the β-subunit of citrate lyase complex (CitE), but the associated α- and γ-subunits needed to form the functional citrate lyase complex appear to be absent in Mtb and, thus, this annotation is likely incorrect (7, 25). Aiming to conduct a comprehensive comparison of Rv2498c in the context of related enzymes, we constructed an amino acid sequence phylogenetic tree of the Pfam HpcH/HpaI aldolase/citrate lyase family (PF03328; *SI Appendix*, Fig. S1) (29). A variety of enzymatic activities have been described for PF03328 members, but the majority fall into 2 classes: aldehyde lyases (EC 4.1.2) and oxoacid lyases (EC 4.1.3); Rv2498c belongs to the latter. Rv2498c and a paralog, Rv3075c, have recently been described by Arora et al. (30) as essential enzymes for Mtb to establish infection in human THP-1 macrophages and guinea pigs. Despite that, substrates and physiologic role of both enzymes remain unknown.

Our phylogenetic analysis suggests that broad substrate specificity and multiple related enzymatic reactions involving analogs of β-hydroxyl-positioned CoA-thioesters (e.g., HMG-CoA, malyl-CoA, β-methylmalyl-CoA, and citramalyl-CoA) are common features in the PF03328 family. Partial phylogenetic coverage, broad substrates specificity, and multiple reactions make functional assignment based on bioinformatics alone unreliable. The substrates and enzymes identified are involved in a variety of pathways including leucine catabolism, itaconate dissimilation, glyoxylate shunt, acetate assimilation, carbon dioxide fixation, C1 assimilation via the serine cycle and via the ethylmalonyl-CoA pathway, C2 assimilation via the ethylmalonyl-CoA pathway, and the methylaspartate pathway (20, 31–34).

### Rv2498c Is Not an (*S*)-Citryl-CoA Lyase.

The (*S*)-citryl-CoA lyase activity initially attributed to Rv2498c was directly tested using an ultraviolet-visible (UV-Vis) high-performance liquid chromatography (HPLC)-based assay. We synthesized (*S*)-citryl-CoA from inactivated citrate lyase as previously described by Buckel et al. (35) and confirmed that Rv2498c is unable to cleave (*S*)-citryl-CoA (*SI Appendix*, Fig. S2). In strict agreement with our results using recombinant enzyme, we found that (*S*)-citryl-CoA is readily hydrolyzed using cell-free protein extracts (CFPEs) derived from both Mtb H37Rv (parent) and from an *rv2498c*-knockout (Δ*rv2498c*) strain (*SI Appendix*, Fig. S2), confirming that Rv2498c is not the enzyme responsible for (*S*)-citryl-CoA hydrolysis.

### Rv2498c Is an (*S*)-Citramalyl-CoA Lyase.

**Fig. 1.**
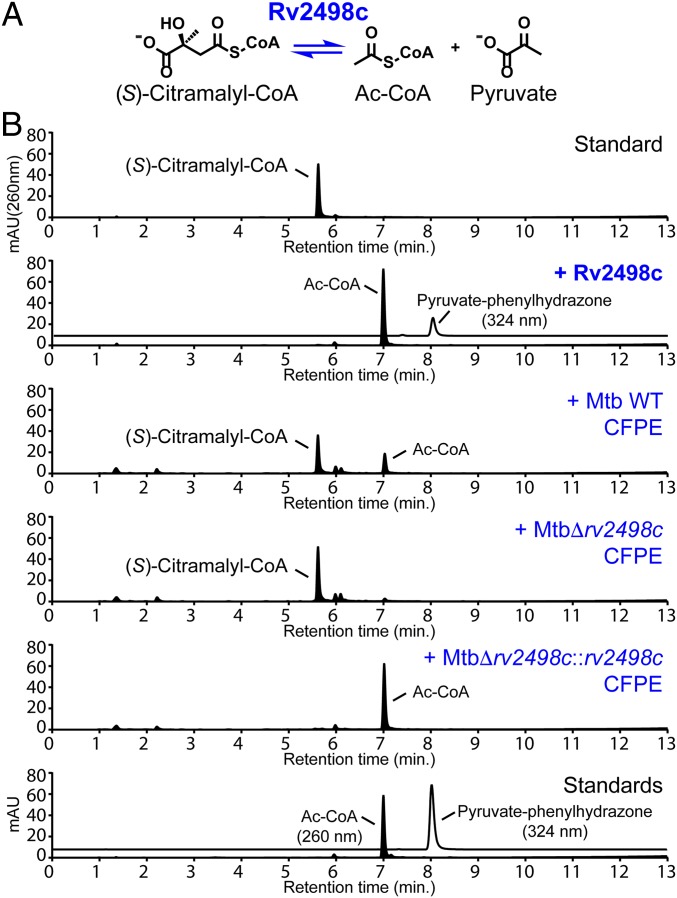
Rv2498c and Mtb CFPE degrade (*S*)-citramalyl-CoA. (*A*) Rv2498c (*S*)-citramalyl-CoA lyase reaction scheme. (*B*) HPLC chromatograms of (*S*)-citramalyl-CoA incubated with or without recombinant Rv2498c or Mtb CFPEs, and standards for Ac-CoA and pyruvate-phenylhydrazone. Pyruvate was derivatized with phenylhydrazine for UV-Vis detection at 324 nm. Degradation of (*S*)-citramalyl-CoA is not observed in the Δ*rv2498c* CFPE chromatogram, indicating that Rv2498c is needed for the activity.

Motivated by the results of the phylogenetic analysis, we tested whether (*S*)-citramalyl-CoA is a substrate for Rv2498c by UV-Vis HPLC. (*S*)-Citramalyl-CoA was found to be a substrate for Rv2498c. Rv2498c catalyzed the carbon–carbon bond cleavage of (*S*)-citramalyl-CoA to form pyruvate and Ac-CoA (*k*_cat_*/K*_m_ = 2.3 × 10^5^; Fig. 1*A* and Table 1). The identity of Rv2498c reaction substrate and products was confirmed by comparing HPLC retention times and mass to charge ratios (*m*/*z*) to those of standards: (*S*)-citramalyl-CoA, Ac-CoA, and pyruvate (Fig. 1*B* and *SI Appendix*, Fig. S3).

**Table 1.** Catalytic properties of recombinant β-HAC lyase and malate synthase from Mtb

Acyl-CoA	Keto acid	*k*_cat_, s^−1^	*K*_m_, µM	*k*_cat_/*K*_m_, M^−1^s^−1^
Rv2498c: β-Hydroxyacyl-CoA lyase activity				
(*R*)-HMG-CoA	—	36 ± 3	90 ± 22	3.9 × 10^5^
(*S*)-Citramalyl-CoA	—	21 ± 2	75 ± 19	2.3 × 10^5^
Rv2498c: malate/methylmalate synthase activity				
Ac-CoA	Glyoxylate	0.15 ± 0.04	2,124 ± 200	6.9 × 10^1^
Pro-CoA	Glyoxylate	0.08 ± 0.03	729 ± 163	1.1 × 10^2^
Rv1837c: malate synthase activity				
Ac-CoA	Glyoxylate	76 ± 4	45 ± 9	1.7 × 10^6^

To validate the results obtained using purified recombinant Rv2498c, we investigated the carbon–carbon cleavage of (*S*)-citramalyl-CoA using CFPEs from parent, Δ*rv2498c*, and an *rv2498c-*complemented strain where the *rv2498c* gene is present elsewhere in the chromosome (Δ*rv2498c*::*rv2498*c). The results using CFPEs were consistent with the results using recombinant Rv2498c, i.e., parent strain and complement strain CFPEs, but not Δ*rv2498c* strain CFPE, degraded (*S*)-citramalyl-CoA (Fig. 1*B*).

### Rv2498c Is an (*R*)-HMG-CoA Lyase.

**Fig. 2.**
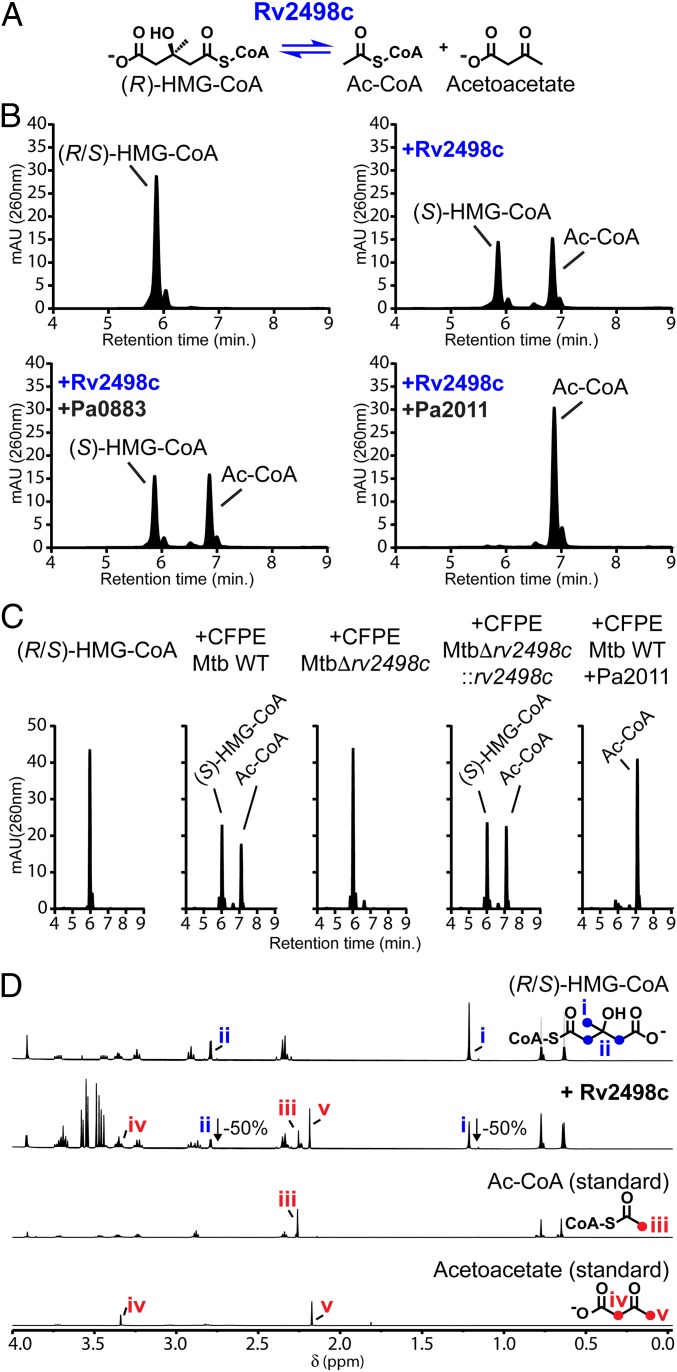
Rv2498c is a stereospecific lyase that cleaves (*R*)-HMG-CoA to produce Ac-CoA and acetoacetate. (*A*) Rv2498c (*R*)-HMG-CoA lyase reaction scheme. (*B*) HPLC chromatograms of HMG-CoA racemic mixture incubated with recombinant Rv2498c. The (*R*)-HMG-CoA lyase stereospecificity of Rv2498c is revealed when adding to the reaction either (*R*)-HMG-CoA-specific lyase Pa0883 or (*S*)-HMG-CoA-specific lyase Pa2011. Rv2498c with Pa0883 only consumed half of HMG-CoA, while Rv2498c with Pa2011 consumed all HMG-CoA. (*C*) HPLC chromatograms of HMG-CoA incubated with Mtb CFPEs with or without (*S*)-HMG-CoA-specific lyase Pa2011. Degradation of (*R*)-HMG-CoA is not observed in the Δ*rv2498c* CFPE chromatogram, indicating that Rv2498c is needed for the activity. (*D*) Comparison between the ^1^H NMR spectra of HMG-CoA incubated with or without recombinant Rv2498c and standards for Ac-CoA and acetoacetate. Peaks assigned to Ac-CoA –CH_3_ (*iii*) group and acetoacetate –CH_3_ (*v*) and –CH_2_– (*iv*) groups are only observed in the spectrum with Rv2498c.

Our phylogenetic analysis also prompted us to screen a diverse panel of commercially available CoA-thioesters (*SI Appendix*, Table S1). In this screen, incubation of Rv2498c with HMG-CoA generated a product with a retention time similar to Ac-CoA and consumed exactly half of the HMG-CoA racemic mixture, suggesting absolute stereospecificity (*k*_cat_*/K*_m_ = 3.9 × 10^5^; Table 1, Fig. 2 *A*–*C*, and *SI Appendix*, Fig. S4); the stereoisomer (*S*)-HMG-CoA is a known metabolic intermediate in the leucine catabolic pathway, which is incompletely annotated in Mtb (36). To unambiguously determine the stereospecificity of Rv2498c for HMG-CoA, we eliminated either the (*R*)- or (*S*)-isomer of HMG-CoA from the reaction mixture by taking advantage of the known stereospecific HMG-CoA lyases from *Pseudomonas aeruginosa*, PA0883 and PA2011 (20). Interestingly, we found that Rv2498c is a lyase specific for the (*R*)-HMG-CoA isomer. Based on the observation that Ac-CoA is one product, the other likely product of C-C bond cleavage is acetoacetate (AAc), which is not visible in our UV-Vis HPLC assay. To directly detect the formation of AAc, we analyzed the reaction products by ^1^H-Nuclear Magnetic Resonance (NMR) spectroscopy and observed the formation of Ac-CoA and AAc from half of the (*R*/*S*)-HMG-CoA in the reaction mixture (Fig. 2*D*). These results demonstrated the stereospecific (*R*)-HMG-CoA lyase activity of Rv2498c.

We also investigated the stereochemical course of HMG-CoA degradation in Mtb CFPE. Consistent with the results obtained using recombinant Rv2498c, parent and complement strains CFPEs, but not Δ*rv2498c* CFPE, degraded (*R*)-HMG-CoA (Fig. 2*C*). These results (*i*) confirmed the (*R*)-specific stereochemical course of HMG-CoA degradation in Mtb, (*ii*) demonstrated that Rv2498c is both necessary and sufficient for the breakdown of the (*R*)-HMG-CoA, and (*iii*) suggested that (*R*)-HMG-CoA is the only isomer present in Mtb.

### Rv2498c Also Displays Weak Malate/Methylmalate Synthase Activity In Vitro.

Unexpectedly, Rv2498c was also found to catalyze the hydrolysis of the thioester bond of (*S*)-malyl-CoA and β-methylmalyl-CoA in vitro (*SI Appendix*, Fig. S5). (*S*)-Malyl-CoA is the in situ condensation reaction product of glyoxylate and Ac-CoA in the glyoxylate shunt, while β-methylmalyl-CoA is the condensation product of glyoxylate and propionyl-CoA in the 3-hydroxypropionate pathway of CO_2_ assimilation. Rv2498c malate/methylmalate synthase activity was observed in vitro using Ac-CoA and propionyl-CoA and an excess quantity of glyoxylate. We determined the kinetic parameters for the reaction and found that Rv2498c is not catalytically comparable to the bona fide malate synthase GlcB (Rv1837c), and these activities are much slower than the reactions with (*R*)-HMG-CoA or (*S*)-citramalyl-CoA (Table 1) (37, 38). The methylmalate synthase activity is not expected to be relevant in vivo as Mtb is not known to possess a 3-hydroxypropionate pathway (31).

From our in vitro results, we have shown that Rv2498c has β-hydroxyl-acyl-CoA lyase and thioesterase activities, and that (*R*)-HMG-CoA, (*S*)-citramalyl-CoA, (*S*)-malyl-CoA, and β-methylmalyl-CoA, but not (*S*)-citryl-CoA, are Rv2498c substrates. These results unambiguously establish that Rv2498c is not a CitE.

### Rv2498c Participates in Itaconate Dissimilation and l-leucine Catabolism in Mtb.

**Fig. 3.**
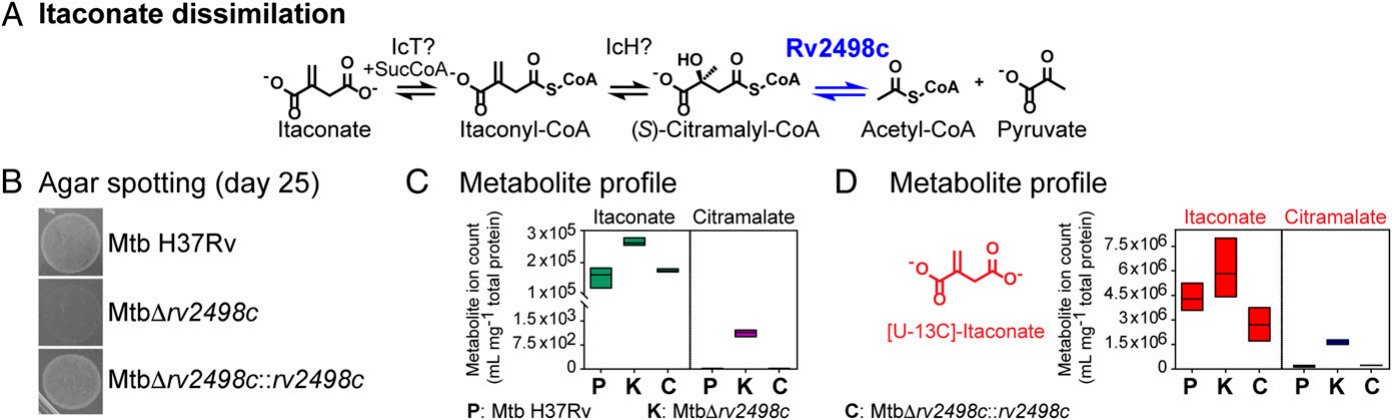
Rv2498c participates in itaconate dissimilation in Mtb. (*A*) The proposed pathway for itaconate dissimilation. (*B*) Agar medium Mtb spotting culture after 25 d on 10 mM itaconate as the sole carbon source. Mtb Δ*rv2498c* failed to grow on itaconate compared with parent and complement strains. The results are representative of 3 independent experiments. Metabolite profile of Mtb filter culture after 17-h exposure to agar medium with 10 mM itaconate (*C*) or 15 mM [13C5]-itaconate (*D*) as the sole carbon source. The metabolite profile shows the accumulation of itaconate/M+5 and citramalate/M+5 (from hydrolyzed citramalyl-CoA) in the Mtb Δ*rv2498c* metabolite extract. The data are shown as mean values ± SD from 3 independent experiments.

**Fig. 4.**
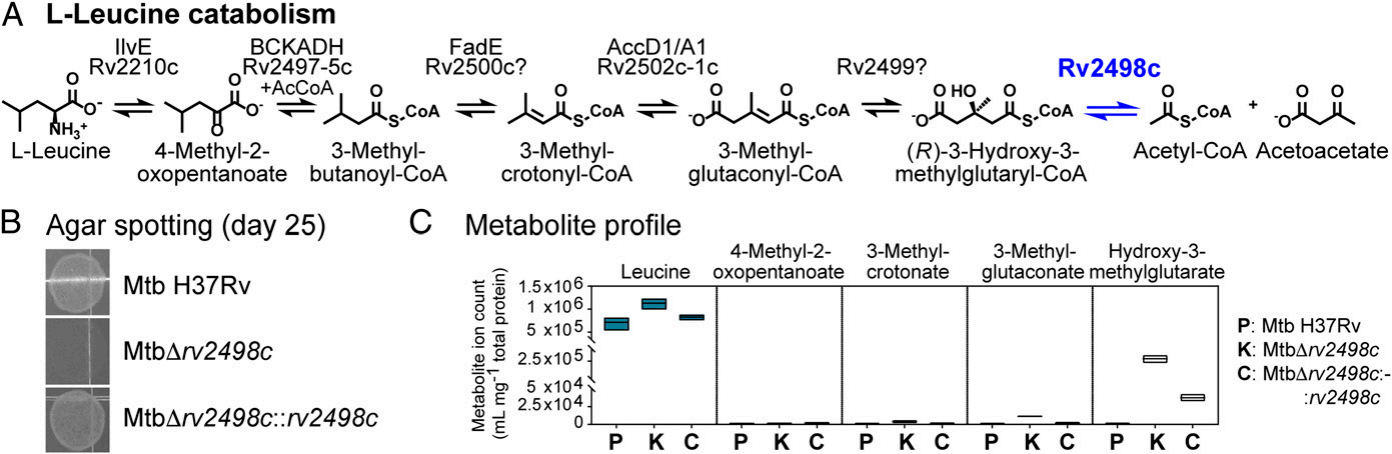
Rv2498c participates in l-leucine catabolism in Mtb. (*A*) The proposed pathway for l-leucine catabolism. (*B*) Agar medium Mtb spotting culture after 25 d on 10 mM l-leucine as the sole carbon source. Mtb Δ*rv2498c* failed to grow on l-leucine compared with parent and complement strains. The results are representative of 3 independent experiments. (*C*) Metabolite profile of Mtb filter culture after 17-h exposure on 10 mM l-leucine as the sole carbon source agar medium. The metabolite profile shows the accumulation of leucine, methylcrotonate (hydrolyzed product), methylglutaconate (hydrolyzed product), and HMG (from hydrolyzed HMG-CoA) in the Mtb Δ*rv2498c* metabolite extract. The data are shown as mean values ± SD from 3 independent experiments.

We interrogated the role of Rv2498c in Mtb metabolism by comparing growth and metabolic profiles of Δ*rv2498c*, parent, and Δ*rv2498c*::*rv2498*c complement strains. These 3 strains were cultured in chemically defined media of composition similar to Middlebrook 7H10 agar medium but with a single carbon source present. In accordance to our substrate specificity results using recombinant Rv2498c and CFPEs, we hypothesized that Rv2498c could be involved in itaconate dissimilation, a process that involves (*S*)-citramalyl-CoA formation (Fig. 3*A*), and in l-leucine catabolism, in which (*R*)-HMG-CoA is a catabolic intermediate (Fig. 4*A*).

A growth defect was observed for Δ*rv2498c* compared with parent and complemented strains on agar medium in the presence of itaconate as the sole carbon source (Fig. 3*B*). A pathway for itaconate degradation has not been described for Mtb, but in mammals and in some bacteria, it is thought to proceed via activation of itaconate to itaconyl-CoA, stereospecific hydration to form (*S*)-citramalyl-CoA, and C-C bond cleavage to form pyruvate and Ac-CoA (Fig. 3*A*) (20–23). Consistent with the existence of this pathway in Mtb and with the involvement of Rv2498c in itaconate degradation, we observed accumulation of citramalate, the hydrolysis product of citramalyl-CoA, in the Δ*rv2498c* strain when grown in itaconate as the sole carbon source, as observed using liquid chromatography-mass spectrometry (LC-MS) (Fig. 3*C*). To unambiguously demonstrate that the observed citramalate is directly derived from itaconate, we employed (U)-^13^C-itaconate as the sole carbon source. Confirming our hypothesis, the citramalate accumulating under these conditions was universally labeled (M+5) (Fig. 3*D*).

Furthermore, consistent with a role of Rv2498c in l-leucine metabolism, no growth was observed on agar medium for the Δ*rv2498c* strain in the presence of l-leucine as the sole carbon source compared with parent and complemented strains on agar medium (Fig. 4*B*). We inferred that this growth deficiency was likely the outcome of the absence of HMG-CoA lyase activity, resulting in the accumulation of HMG, limited production of Ac-CoA, and/or altered branched-lipid metabolism. Accordingly, in the presence of l-leucine as the sole carbon source, Δ*rv2498c*, but not the parent or the complemented strains, accumulated HMG, as well as other leucine catabolism intermediates such as methylcrotonate, methylglutaconate, and hydroxymethylglutarate, as observed using LC-MS (Fig. 4*C*).

These in vivo experiments corroborated our in vitro and ex vivo biochemical data and offer further evidence that Rv2498c is a bifunctional enzyme participating in itaconate dissimilation and leucine catabolism in Mtb.

### The MtbΔ*rv2498c* Strain Is Attenuated in a Mouse Aerosol Infection Model.

To test the impact of *rv2498c* deletion during infection, we carried out a low-dose aerosol infection of C57BL/6J mice, a mouse strain widely used in experimental TB research studies. The MtbΔ*rv2498c* strain resulted in at least one log_10_ reduction in colony forming units in the lung at days 28, 84, and 112 after infection compared with the parent strain (*SI Appendix*, Fig. S6). The attenuation observed for the Δ*rv2498c* strain suggests that one or more of the activities associated with Rv2498c have a significant negative impact on the fitness of Mtb during experimental infection.

### Rv2498c (*S*)-Citramalyl-CoA Bound Structure Reveals the Molecular Basis for Stereospecificity.

To investigate the molecular basis of Rv2498c substrate stereospecificity for the carbon–carbon bond, we determined the structures of Rv2498c in a variety of liganded states by X-ray crystallography (*SI Appendix*, Table S2). All structures obtained showed the same trimeric arrangement of protomers previously described for the unliganded structure and for the oxaloacetate- and Mg^2+^-bound structure (25). In contrast to the previously reported structures, the C terminus (50 residues) is well ordered in our structures, forming an α-helix/β-hairpin/α-helix motif that packs against the surface of the neighboring protomer, capping its active site (*SI Appendix*, Fig. S7*A*). The fact that the C terminus is ordered in these ligand-bound structures is consistent with earlier observations that the position and organization of the C terminus might depend on the occupancy of the active site (*SI Appendix*, Fig. S7*B*) (33).

Rv2498c has no sequence and modest structural similarity to the characterized family of TIM barrel (*S*)-HMG-CoA lyases that include human HMG-CoA lyase (Protein Data Bank [PDB] ID code 3MP5) and the bacterial lyases from *Brucella melitensis*, *Bacillus subtillis*, and *P. aeruginosa* (PDB ID codes 1YDN, 1YDO, and 2FTP) (39). Instead, the 3 most closely related ligand-bound structures to Rv2498c are the human citramalyl-CoA lyase CLYBL (PDB ID code 5VXO) and the bacterial l-malyl-CoA/β-methylmalyl-CoA lyases from *Rhodobacter sphaeroides* (PDB ID code 4L9Y) and from *Chloroflexus aurantiacus* (PDB ID code 4L80) with RMSDs of 2.35 (over 261 Cα atoms), 2.43 (over 266 Cα atoms), and 2.65 (over 266 Cα atoms), respectively (14, 33, 40, 41). These 3 proteins were crystallized with propionyl-CoA in their active sites.

**Fig. 5.**
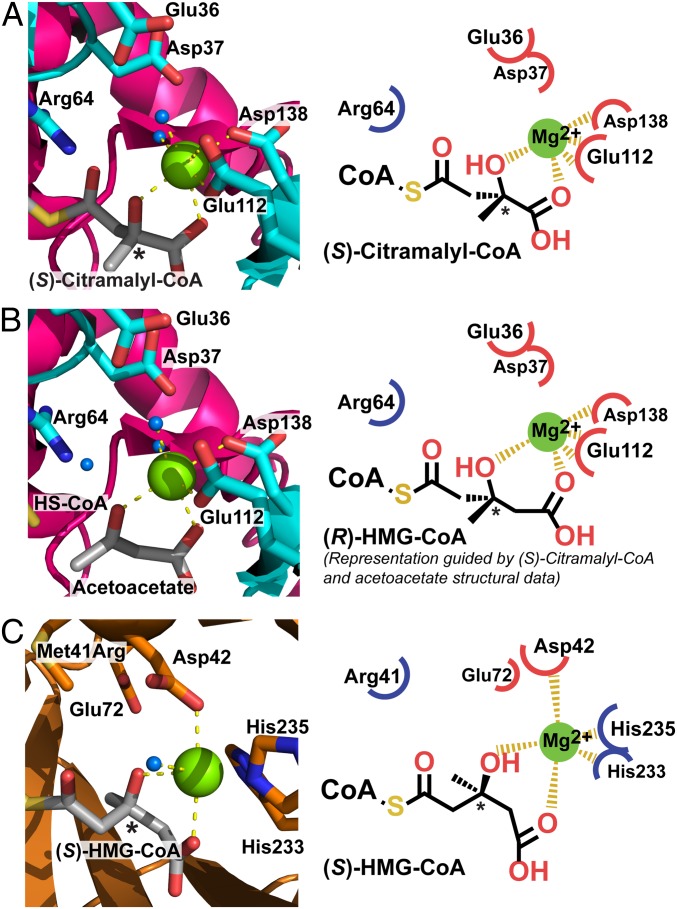
Ligand-bound X-ray 3D structures of Rv2498c reveal that metal coordination is the basis of stereoselectivity. (*A*) Close-up view of the catalytic site and a representation of (*S*)-citramalyl-CoA-bound Rv2498c (PDB ID code 6AQ4). The β-hydroxyl group chelation of the Mg^2+^ ion favors the *S*-conformation of citramalyl-CoA. (*B*) Close-up view of the catalytic site of acetoacetate-bound Rv2498c (PDB ID code 6AS5), and a representation of (*R*)-HMG-CoA in Rv2498c was modeled based on the reaction substrate (*S*)-citramalyl-CoA (*A*) and the reaction product acetoacetate. Acetoacetate, the HMG-CoA carbon–carbon cleavage product retains the keto-acid conformation. (*C*) Close-up view of the catalytic site and a representation of human (*S*)-HMG-CoA lyase R41M mutant (PDB ID code 3MP5) bound to (*S*)-HMG-CoA is shown for comparison. Ribbon and stick representations were generated using PyMOL and ChemDraw. Atoms are colored according to the CPK coloring scheme. *, a chiral carbon center. Blue spheres represent water molecules and green spheres represent Mg^2+^ ions.

The Rv2498c structures presented here are complexes with (*S*)-citramalyl-CoA (PDB ID code 6AQ4) and with acetoacetate:CoA (PDB ID code 6AS5). The CoA moiety binds in a deep cleft at the base of which resides the active site Mg^2+^ ion. The Mg^2+^ ion is coordinated by the 3-hydroxyl group and by an unidentate interaction with the terminal carboxylate of the ligand, as well as by Glu112, Asp138, and 2 water molecules (Fig. 5 *A* and *B*). The carboxylate of the substrate is further positioned by polar interactions with the backbone NH atoms of Ala136, Glu137, and Asp138, while the 3-hydroxyl group lies close to Arg64. The 3-methyl group of the ligand contacts a hydrophobic surface formed by Gly135 and the side-chain atoms of Met133 and Val181 (*SI Appendix*, Fig. S8). Of note, the structure with (*S*)-citramalyl-CoA bound was obtained from crystals that were soaked with pyruvate and Ac-CoA, therefore indicating that the conformation of Rv2498c in the crystals is catalytically active.

Because the citramalyl group observed in the active site was in the *S* configuration, which corresponds to the *R* configuration at the 3-position of HMG-CoA (*SI Appendix*, Fig. S9), we modeled the mode of binding of (*R*)-HMG-CoA to further understand the molecular basis for the (*S*)-HMG-CoA lyase associated with mammalian l-leucine catabolism. In this case, the additional methylene unit (C4) found in HMG-CoA is readily accommodated in the active site and can support a less distorted octahedral coordination of the Mg^2+^ ion (the bite angle of the citramalyl group is ∼70°; the modeled HMG moiety bite angle is ∼85°). Likewise, Molprobity calculations revealed shape and charge complementarity between the protein and modeled substrate, consistent with the active site supporting energetically favorable interactions with (*R*)-HMG-CoA (42). Attempts to model (*S*)-HMG-CoA into the active site generated electrostatically unfavorable interactions. Interestingly, with respect to the human (*S*)-HMG-CoA lyase (Fig. 5*C*, PDB ID code 3MP5), the differential positioning of the Mg^2+^ ion coordination with conserved Asp and Glu residues in the active site contributes to the stereoselectivity between the HMG-CoA stereoisomers.

## Discussion

Mtb is highly adapted to the human host and is physiologically resilient. We show that Mtb can dissimilate itaconate, a macrophage metabolite produced during host inflammatory response to fight infection (12, 14, 15). In contrast to the established antimicrobial character of this molecule, itaconate is present in some prokaryotes (12, 14–17, 20, 21, 23, 43, 44). Moreover, our results suggest that Mtb can catabolize itaconate via a noncanonical dissimilation pathway. Itaconate dissimilation in Mtb involves the bifunctional enzyme Rv2498c, which cleaves (*S*)-citramalyl-CoA producing Ac-CoA and pyruvate, effectively using a host-derived antibacterial molecule as a nutrient source.

We show that Mtb catabolizes l-leucine via an unprecedented use of (*R*)-HMG-CoA rather than the commonly attributed (*S*)-HMG-CoA isomer. Mtb lacking Rv2498c appears to suffer the same fate as humans with HMG-CoA lyase-deficiency, the accumulation of HMG and keto acids from the leucine catabolic pathway (45). In addition to l-leucine, we tested l-valine and l-isoleucine (the 2 other branched-chain amino acids) and observed no differences in growth phenotypes between the parent and the Δ*rv2498c* strains. These results agree with the known differences in the catabolism of these 3 amino acids; HMG-CoA is produced during l-leucine degradation and not a shared metabolic intermediate in the catabolism of branched-chain amino acids.

We also found that Rv2498c could function as a malate/methylmalate synthase, albeit with low efficiency, by catalyzing an aldol condensation followed by thioester hydrolysis of (*S*)-malyl-CoA or β-methylmalyl-CoA, resulting in the formation of malate from glyoxylate and Ac-CoA, or methylmalate from glyoxylate and propionyl-CoA, respectively. The physiologic role for Rv2498c as a putative synthase/thioesterase is unclear as Mtb possesses a bona fide malate synthase (GlcB, Rv1837c) (38, 46). However, the first step in the glyoxylate shunt is also catalyzed by 2 apparently redundant isocitrate lyases (Rv0467 and Rv1915-Rv1916). Although there is no known metabolic pathway in Mtb which uses β-methylmalyl-CoA, Rv2498c could act to detoxify glyoxylate and propionyl-CoA from odd chain lipid catabolism under glyoxylate shunt metabolic state (46). Nonetheless, such potential redundancy might be metabolically advantageous if the enzymes display different kinetic or regulatory properties in vivo. It is also possible that multifunctional enzymes, such as Rv2498c, allow for circumvention in the presence of enzyme inhibitors of the canonical enzymes (31, 46, 47).

Our crystal structures for full-length Rv2498c shed light into the molecular basis of substrate specificity and stereoselectivity for (*S*)-citramalyl-CoA and revealed how the C-terminal domain interacts directly with the neighboring CoA substrate, therefore playing an important role in substrate binding selectivity. We suggest that Rv2498c carbon–carbon cleavage of (*S*)-citramalyl-CoA follows a mechanism similar to that proposed for (*S*)-HMG-CoA lyase described by Fu et al. (48) (*SI Appendix*, Fig. S10) and that the difference in the (*S*)- and (*R*)-stereospecificity for HMG-CoA can be partially attributed to the β-positioned hydroxyl group of the CoA-thioester coordination with the divalent metal. We observed that the Rv2498c catalytic site has ordered water molecules, suggesting a catalytic mechanism for the carbon–carbon cleavage with water participation by shuttling protons and/or acting as the nucleophile during hydrolysis (33, 48, 49). The lyase reaction likely proceeds via the reversal of standard retro-aldol condensation, as previously suggested (48, 50).

In conclusion, we found Rv2498c, which we renamed as a bifunctional β-HAClyase, in a class of oxoacid lyases with paradoxical features: characterized by mild promiscuity for substrates but absolute discrimination on the substrate stereochemistry. A systematic evaluation of substrate specificity revealed (*R*)-HMG-CoA ∼ (*S*)-citramalyl-CoA >>> (*S*)-malyl-CoA ∼ β-methylmalyl-CoA as substrates for Rv2498c. Our results demonstrate that Mtb possesses the ability to dissimilate itaconate and an (*R*)-specific HMG-CoA l-leucine catabolic pathway. We further elucidated the enzymatic activities of Rv2498c as a β-HAClyase and presented a full-length crystal structures of the protein, which revealed the details of its substrate stereospecificity and the involvement of the C-terminal domain in acyl-CoA binding. Importantly, deletion of *rv2498c* from the Mtb genome led to a defect during murine infection, indicating that one or more of its enzymatic functions are important during infection. Our work further highlights that understanding of even the most well conserved and central metabolic pathways in Mtb is hampered by the prevalence in the genome of experimentally uncharacterized enzymes and enzymatic function database misannotations. Mtb Rv2498c is a striking example of an enzyme that eluded functional characterization for over a decade, highlighting the intricacies and difficulties of enzyme functional assignment.

## Materials and Methods

All biological and chemical reagents were purchased from Sigma-Aldrich or Fisher Scientific, unless stated otherwise. Minimal media of chemically defined formulae were prepared in-house. *pML1335-GFP or pML1357* was a gift from Michael Niederweis (Addgene plasmid 32378; http://www.addgene.org/32378/; RRID:Addgene_32378). A full description of methods for gene cloning, protein expression and purification, phylogenetic analysis, Mtb growth conditions, enzyme assays, murine Mtb aerosol infections, metabolomics, HPLC, LC-UV/MS, and X-ray data collection and analysis are described in *SI Appendix*.

## Supplementary Material

Supplementary File
