# Supplementary material for: An essential bifunctional enzyme in Mycobacterium tuberculosis for itaconate dissimilation and leucine catabolism
Source: Proc Natl Acad Sci U S A. 2019 Jul 18;116(32):15907–13. doi: 10.1073/pnas.1906606116 (PMC6689899; doi:10.1073/pnas.1906606116)
Supplement: Supplementary File [file pnas.1906606116.sapp.pdf]

PNAS
www.pnas.org

Supplementary Information for

An essential bi-functional enzyme in *Mycobacterium tuberculosis* for itaconate dissimilation and leucine catabolism

Hua Wang[a,1], Alexander A. Fedorov[b], Elena V. Fedorov[b], Deborah M. Hunt[a], Angela Rodgers[a], Holly L. Douglas[a], Acely Garza-Garcia[a], Jeffrey B. Bonanno[b], Steven C. Almo[b], Luiz Pedro S. de Carvalho[a,2]

[a]Mycobacterial Metabolism and Antibiotic Research Laboratory, The Francis Crick Institute, London, UK, and [b]Department of Biochemistry, Albert Einstein College of Medicine, New York City, USA.

[1]Present address: Institute of Infection, Immunity and Inflammation, University of Glasgow, Glasgow, G12 8TA.
[2]Correspondence: Luiz.Carvalho@crick.ac.uk

This PDF file includes:

Materials and Methods
Figs. S1 to S10
Tables S1 and S2
References for SI reference citations

www.pnas.org/cgi/doi/10.1073/pnas.1906606116

Materials and Methods

All biological and chemical reagents were purchased from Sigma Aldrich or Fisher Scientific, unless stated otherwise. Minimal media of chemically defined formulae were prepared in-house. *pML1335-GFP or pML1357* was a gift from Michael Niederweis (Addgene plasmid # 32378; http://n2t.net/addgene:32378; RRID:Addgene_32378)(1).

Cloning, expression and purification of *M. tuberculosis* H37Rv Rv2498c, *Pseudomonas aeruginoa* PA0883 and PA2011, and *Chloroflexus aurantiacus* MMC lyase.

The *rv2498c* gene was amplified from Mtb H37Rv genomic DNA by PCR (primers: 5'-GCCATATGGCACACCACCACCACCACCACATGAACCTGCGTGCCGCCGG-3', 5'-GCAAGCTTTCATTCGGAGGTGGCTTCCC-3'). The PCR product was isolated and ligated into the *NdeI* and *HindIII* sites of pET-23a(+) coding for a N-terminal His_6 tag. The construct was verified by Sanger sequencing.

Gene sequences for *PA0883*, *PA2011*, and *MMC lyase* were codon-optimized for *Escherichia coli* (*E. coli*) expression, synthesised, and then cloned (*NdeI-BamHI*) into pET-16b by GenScript (Piscataway, NJ).

E. coli BL21(DE3) (New England BioLabs) competent cells were transformed with corresponding plasmids following the manufacture transformation protocol. A single colony was used to prepare a starter culture in LB medium containing 100 µg of carbenicillin, or 50 µg of kanamycin, per mL, and incubated at 37 °C overnight. A freshly prepared 1-L LB medium with selected antibiotic was inoculated with 100 µL of starter culture. The culture was grown to an optical density (OD_{600}) of 0.6-8 and then cooled to 22 °C, followed by the addition of 1 mM isopropyl-thiogalactopyranoside to induce the expression. The culture was incubated for an additional 12 hr at 22 °C, 180 rpm, and then the cells were harvested after centrifugation. The cell pellet was either directly lysed and used for protein purification or stored at -80 °C until use.

The cell pellet was suspended in buffer (50 mM HEPES, 300 mM NaCl, 5 mM $MgCl_2$, pH 7.5) containing lysozyme (hen egg white), DNase (TURBO, Thermo Fisher Scientific Inc.), and Complete® protease inhibitor cocktail EDTA-free (Pierce), and then stirred on ice for 2 hr followed by sonication 3x 30 seconds on ice. The cell lysate was centrifuged at 20,000 rpm, 4 °C, for 1 hr. The clear supernatant was incubated with Ni-NTA resin that has been equilibrated with buffer containing 10 mM imidazole, at 4 °C for 3 hr. The resin slurry was then transferred to a glass Econo-column (Bio-Rad), washed with the buffer, and then eluted by a step-wise discontinuous imidazole gradient up to 500 mM imidazole. The fractions were analysed by SDS-PAGE. The protein fractions were pooled and concentrated using Vivaspin, and then purified by size-exclusion chromatography (HiLoad 16/600 Superdex 200 pg, GE Healthcare). The protein fractions were analysed by SDS-PAGE, and the protein fractions were pooled and concentrated using Vivaspin. The protein concentration was determined by BCA assay (Pierce). The proteins were stored at -20 °C as 50% glycerol stock or -80 °C as 25% glycerol stock.

Cloning, expression and purification of *M. tuberculosis* H37Rv malate synthase G (GlcB; Rv1837c)

Platinum SuperFi DNA Polymerase (ThermoFisher) was used to amplify the coding region of *glcB* from *M. tuberculosis* H37Rv genomic DNA using the primers 5'-AGAACCTGTACTTCCAATCCATGACAGATCGCGTGTCGGTGGGCA-3' and 5'-GATCCGTATCCACCTTTACTGTTAGCGGGCCGCATCGTCACCG-3'. The PCR product

was cloned by isothermal assembly (HiFi DNA Assembly, NEB) into a modified pET28a vector (Merck) coding for an N-terminal His_6 tag followed by a TEV protease cleavage site. The construct was verified by Sanger sequencing.

E. coli BL21 Star (DE3) (ThermoFisher) cells were transformed with the construct. A single colony was used to inoculate 10 mL of lysogeny broth. The cell pellet obtained after overnight incubation (37 °C and 200 rpm) was diluted 50X into autoinduction medium (ZYM-5052/60 μg/mL kanamycin). Cells were grown for 4 hours at 37 °C followed by 20 hours at 18 °C.

Cells were harvested by centrifugation, resuspended in 50 mM Tris, 300 mM NaCl, 10 mM imidazole, pH 8.0 and lysed by sonication. The cell lysate was clarified by centrifugation and the overexpressed protein was purified by gravity-driven Ni-NTA (His-Bind Superflow, Merck) followed by size-exclusion chromatography (HiLoad 16/600 Superdex 200 pg, GE Healthcare) into HEPES 50 mM, NaCl 300 mM, pH 7.5

Phylogenetic analysis

A seed multiple sequence alignment (MSA) of selected members of the HpcH_HpaI aldolase/citrate lyase family (PF03328) was built based on an alignment of the structures available in the PDB.(2) Structures were visualised and edited using Chimera and the multiple structural alignments was computed by Mustang.(3, 4) The PF03328 sequences in the full-alignment were downloaded from PFAM and aligned to the seed MSA using MAFFT E-INS-I-add option.(5) Partial and highly divergent sequences were removed and redundancy was reduced to 90% ID or less using Jalview.(6) The model of protein evolution used was LG+I+G+F with α of 1.64 and p-inv of 0.01, as selected by Prottest 3.4.(7) Bootstrap repeats and consensus trees were generated with PHYLIP.(8) Pairwise distances were calculated with TREEPUZZLE *via* the puzzleboot script.(9) Distance trees were calculated with BIONJ.(10) For the acyl-CoA lyase tree, sequences were aligned with MUSCLE and a maximum-likelihood tree was calculated with PhyML.(11, 12) Trees were drawn with Dendroscope 2.7.(13)

Mycobacteria and culture conditions

The experiments in the study used pathogenic *Mycobacterium tuberculosis* (Mtb) strain, *Mtb*-H37Rv, which requires Containment level 3 facility. All mycobacteria work was carried out in the Containment level 3 facility. Mtb cultures were typically grown at 37 °C in Middlebrook 7H9 medium (Sigma) supplemented with 10% albumin-dextrose-catalase supplement (ADC, Sigma) and 0.05% Tyloxapol (Sigma) or on Middlebrook 7H11 agar medium (Sigma) supplemented with 10% oleic acid-albumin-dextrose-catalase supplement (OADC, Sigma). Minimal medium is modified-7H9 or -7H10 medium without glycerol, L-glutamic acid, oleic acid, and dextrose. Chemically defined medium is modified-7H9 or -7H10 minimal medium with 10mM of test substrates (*e.g.* L-leucine, itaconate, L-valine, L-isoleucine, acetate, oleate, pyruvate… *etc*.).

Mtb spotting culture

The spotting culture is essentially the conventional bacteria growth on agar medium without streaking the bacteria. Mtb liquid cultures were grown in 7H9 medium supplemented with Tyloxapol and ADC to OD_{600} of 1. Serial dilutions of 1:5. 2 μL of the OD_{600} of 1 and the diluted cultures were spotted on chemically defined-7H10 medium with test compounds (*e.g.* L-leucine, itaconate, L-valine, L-isoleucine, acetate, oleate, pyruvate… etc.). The plates were incubated at 37 °C. Growth phenotypes were observed, and images were taken on day 25 post spotting.

Cell-free protein extract preparation

Mtb liquid culture was grown at 37 °C in 7H9 medium supplemented with Tyloxapol and ADC to OD_{600} of 1. The cell pellet was collected after centrifugation at 2,000 rpm for 10 min at 4 °C, and then washed with HEPES (50 mM, pH 7.5, 5 mM $MgCl_2$). The washed cell pellet was resuspended in the HEPES buffer containing cOmplete™, EDTA-free Protease Inhibitor Cocktail (Roche) and transferred to O-ring screw cap tube with glass beads (150-212 μm, acid washed, Sigma), and ribolysed at 2 x 30 sec with cooling in between the two cycles. The clear supernatant was collected after centrifugation at 13,000 rpm for 10 min at 4 °C, and then filtered twice through 0.2 μm membrane. The total protein concentration was determined by BCA assay (Pierce).

Syntheses of CoA-thioesters

Citramalyl-CoA, malyl-CoA, and β-methylmalyl-CoA were synthesised enzymatically with MMC lyase, as described by Zarzycki *et al.*(14) Citryl-CoA was synthesised enzymatically with inactivated citrate lyase from *Klebsiella pneumoniae* (Sigma), as described by Buckel *et al.*(15) The CoA-thioesters were analysed by HPLC, and their identities were confirmed by high-resolution mass spectrometry.

Murine aerosol *M. tuberculosis* infections

C57BL/6J (WT) mice were bred and maintained under specific pathogen-free conditions at The Francis Crick Institute, Mill Hill Lab. Animal studies and breeding were approved by the Francis Crick Institute ethical committee and performed under U.K Home Office project license PPL 70/8045. Infections were performed in the category 3 animal facility. For mouse infections, *M. tuberculosis* strains were cultured in Middlebrook 7H9 broth containing ADC to an OD_{600} of 0.6. From this, an infection sample was prepared to enable delivery of 100 colony forming units (CFUs)/mouse lung using a modified Glass-Col aerosol infection system. Infection was monitored by assessing homogenised lungs from infected mice at defined periods post-infection. Bacterial CFUs were determined by plating serial dilutions of homogenates on duplicate Middlebrook 7H11 containing OADC. Colonies were counted 2-3 weeks after incubation at 37 °C. The data at each time point are the means of 5 mice/group +/- SEM.

Filter culture and metabolite preparation

The filter culture followed the protocol as described by Carvalho *et al.*, with minor modifications.(16) Mtb was cultivated in 7H9 medium supplemented with Tyloxapol and ADC at 37 °C to reach an OD_{600} of 1. A total of 1-mL of the liquid culture was transferred to a membrane disc under vacuum to collect the cells on the membrane. The membrane disc containing the cells was transferred on 7H10 medium supplemented with OADC and incubated for 5-6 days until sufficient cell mass has accumulated. The membrane disc containing the cell mass was then transferred to a conditioned-7H10 minimum medium supplemented with relevant test substrate and incubated for an additional 17 hr maximum to avoid doubling of the cells. After the 17 hr exposure, the cells were scrapped and transferred to an O-ring screw cap tube with glass beads (150-212 μm, acid washed, Sigma), with the addition of 0.5-0.7 mL cold acetonitrile:methanol:water (2:2:1) and ribolysed for 2 x 30 sec with cooling between the two cycles. The clear supernatant was collected after centrifugation at 13,000 rpm for 10 min at 4 °C and filtered twice through 0.2 μm membrane. The filtered supernatant was analysed by LC/MS or stored at -80 °C until further use.

LC-MS metabolomics

The clear supernatants from metabolite preparation were directly analysed by the LC-MS method described by Larrouy-Maumus *et al.*(17) Briefly, an Agilent 1200 LC system

consisting of a solvent degasser, binary pump, temperature-controlled auto-sampler and temperature-controlled column compartment equipped with a Cogent Diamond Hydride Type C silica column (150 mm × 2.1 mm; dead volume 315 μL) was used for liquid chromatography. The flow rate was 0.4 ml/min. Solvent A was 0.2% acetic acid in water and solvent B was 0.2% acetic acid in acetonitrile. The gradient was as follows: 0-2 min 85%B; 2-3 min to 80%B; 3-5 min 80%B; 5-6 min to 75%B; 6-7 min 75%B; 7-8 min to 70%B; 8-9 min 70%B; 9-10 min to 50%B; 10-11 min 50%B; 11-11.1 min to 20%B; 11.1-14 min hold 20%B.(18)

An Agilent Accurate Mass 6230 TOF apparatus was used. Dynamic mass axis calibration was achieved by continuous infusion of a reference mass solution using an isocratic pump connected to a multimode ionization source, operated in the positive-ion and negative-ion mode. ESI capillary and fragmentor voltages were set at 3500 V and 100 V, respectively. The nebulizer pressure was set at 40 psi and the nitrogen drying gas flow rate was set at 10 L/min. The drying gas temperature was maintained at 250°C. The MS acquisition rate was 1.5 spectra/sec and *m*/*z* data ranging from 50-1200 were stored. The instrument routinely enabled accurate mass spectral measurements with an error of less than 5 parts-per-million (ppm), mass resolution ranging from 10,000-25,000 over the m/z range of 121-955 atomic mass units, and a 100,000-fold dynamic range with picomolar sensitivity. Data were collected in the centroid mode in the 4 GHz (extended dynamic range) mode.

LC-UV/MS

LC-UV/MS analysis was performed on an Agilent 1290 LC system equipped with a diode array UV detector coupled to an Agilent 6560 Ion Mobility Q-ToF (operated in Q-ToF only mode).

Chromatography was performed using an Agilent Poroshell 120 EC-C18, 2.7 μm, 4.6 x 50 mm column (ambient temperature) and 40 mM ammonium formate, pH 6.8 and acetonitrile as mobile phases A and B respectively. Analytes were eluted using a flow rate of 0.5 mL/min and the following mobile phase gradient: 0 – 1 min, 2% B; 1 – 10 min, 2 – 20% B; 10 – 12 min, 20% B. The system was re-equilibrated to initial conditions for two minutes at the end of each run. The injection volume was 3 μL for all standards and samples. UV detection was carried out at 260 nm.

The Q-ToF was operated in negative-ion mode with electrospray ionisation (ESI) using a dual AJS ESI source. Capillary, nozzle and fragmentor voltages were set at 3000 V, 2000 V and 380 V respectively. The nebulizer pressure was set at 40 psi and the nitrogen drying gas flow rate was set at 10 L/min. The drying gas temperature was maintained at 200 °C. The sheath gas temperature and flow rate were 350 °C and 11 L/min. The MS acquisition rate was 1 spectra/sec and *m*/*z* data ranging from 50-1700 were stored. Dynamic mass axis calibration was achieved by continuous infusion of a reference mass solution, which enabled accurate mass spectral measurements with an error of less than 5 parts-per-million (ppm).

Enzyme assays

Rv2498c or cell-free protein extract (CFPE) reaction was carried out in buffer containing 50 mM HEPES (pH 7.5), 5 mM $MgCl_2$, and specified concentration of substrate (usually in the 10-50 μM range). Reaction was initiated by the addition of enzyme or CFPE, and incubated at 37 °C for 30 minutes, 2 hours, or overnight. The reactions were terminated on ice and quenched with 1 μL of 1M HCl per 10 μL reaction volume or without quenching, and directly used for HPLC analysis. Typically, the reaction mixture was quenched and centrifuged at 13,000 rpm for 10 min at 4 °C, and the clear supernatants were used for HPLC analysis.

Measurement of enzyme kinetics

The kinetic studies were carried out in buffer containing 50 mM HEPES (pH 7.5), 5 mM $MgCl_2$, a fixed concentration of the enzyme, and specified concentration of substrate (usually 25, 50, 100, 250, 500 μM). The reaction was initiated by the addition of the purified enzyme, with aliquots of the reaction collected at various times and quenched with HCl on ice for each concentration of the substrate to determine the forward initial velocities. The clear supernatant of the quenched samples was collected after centrifugation (13,000 rpm, 10 min, 4 C), and then injected into the HPLC. Substrate and product were measured at 260 nm, and the concentration of the reaction product was determined using an Ac-CoA standard curve. The results were fitted on Michaelis-Menten curve or Lineweaver-Burk plot to determine the V_{max} and K_m. The calculation for HMG-CoA lyase kinetic values took into consideration of the commercial equal molar racemic mixture of (*R*/*S*)-HMG-CoA by dividing the concentration of HMG-CoA substrate by two.

Analytical HPLC

HPLC-based methods were used to directly detect the CoAs with UV absorption at 260 nm. An Agilent 1260 Infinity (Santa Clara, CA) HPLC apparatus was used, equipped with G1311B 1260 QuatPump, G1316A 1260 TCC, G1264C 1260 FC-AS, G1267E 1260 HiP ALS, and G4212B 1260 DAD.

Each CoA-thioester was detected as a single peak after being separated by HPLC with a Poroshell 120 EC-C18, 2.7 μm, 4.6 x 50 mm column (Agilent) using the following elution condition: 1-min isocratic elution at 2% acetonitrile in buffer, followed by a 10-min linear gradient of 2–20% acetonitrile, with 2-min isocratic elution at 20% acetonitrile in buffer, and then back to 1-min isocratic elution at 2% acetonitrile in buffer at a flow rate of 0.5 ml/min. The buffer used was 40 mM ammonium formate, pH 6.8. For more hydrophobic CoA-thioesters, 95% instead of 20% acetonitrile was used for the gradient. When necessary, CoA-thioesters were quantified by a calibration curve generated from the authentic Acetyl-CoA standard (Sigma-Aldrich) stock solutions (5, 15, 25, 50, 100, 150, 250 μM).

Pyruvate derivatization with phenylhydrazine HCl.

Pyruvate derivatization followed the method described by Lange and Mályusz with minor modifications.(19) Briefly, 1.5 molar equivalent of phenylhydrazine was added to the acid quenched CoA crude reaction mixture and incubated for 1 hr at 37 °C. The clear supernatant of the reaction mixture was collected after centrifugation and then injected in the HPLC for analysis. The pyruvate-phenylhydrazone is monitored as 324 nm.

^{1}H Proton nuclear magnetic resonance spectroscopy

Samples were prepared in 10% D_2O or D_2O phosphate buffer (0.1 M, pD = 7.2). Proton nuclear magnetic resonance (δH) spectra were recorded on Bruker Avance III HD 400 (400 MHz), Bruker Avance III 600 (600 MHz), or Bruker Avance III HD 800 (800 MHz). All chemical shifts were quoted on δ-scale in ppm, with residual solvent as internal standard.

Construction of Mtb *rv2498c*KO mutant and complements

The construction of Rv2498c knockout followed methods described by Parish et al.(20) Briefly, an unmarked in-frame deletion of the *Rv2498c* gene was made by amplifying 1.5 kb of flanking sequence from Mtb H37Rv with Phusion Taq (upstream of Rv2498c: 5’-GCAGATCTTCGGCGGCCATCGCGTCGTA-3’, 5’-GCTCTAGAACCTCCGAATGAGGGCGCAG-3’; downstream of Rv2498c: 3’ for: 5’-GCTCTAGAACGCAGGTTCATTGCGCCTC-3’, 5’-

GCAGATCTTGGTACTTGAGGAGCTGGGC-3’), and cloned into PCR4blunt (Invitrogen). The inserts digested with *BglII* and *XbaI*, and the fragments were ligated together using T4 DNA ligase. The ligation product was amplified (5’-GCAGATCTTCGGCGGCCATCGCGTCGTA-3’, 5’-GCTCTAGAACGCAGGTTCATTGCGCCTC-3’), digested with *BglII* and cloned into the *BamH1* site of p2NIL. The *PacI* fragment of pGOAL17 containing the *lacZ* and *sacB* genes was cloned into the *PacI* site of the resulting plasmid to make the final *rv2498c*KO construct plasmid. Competent Mtb H37Rv was electroporated with the knockout construct plasmid, and single crossovers were selected on 7H11 plates containing kanamycin and X-gal. The blue colonies were then streaked on 7H11 plates and then double crossovers were selected on 7H11 plates containing sucrose and X-gal, the resulting white colonies were screened for double crossovers. Southern blot was performed to confirm a knockout of Rv2498c.

For the complement, *rv2498c* gene fragment was amplified from Mtb H37Rv genomic DNA by PCR (5’-AACAGAAAGGAGGTTAATAATGAACCTGCGTGCCGCC-3’, 5’-TTAGCTAAAGCTTATTTAAATTCATTCGGAGGTGGCTTCCCCG-3’) and assembled with pML1335 integrative vector PCR fragment (5’-ATTTAAATAAGCTTTAGCTAATTAATTGGGGACCCTAGAGGTC-3’, 5’-TATTAACCTCCTTTCTGTTAATTAAGCATGCGGATCGT-3’) by Gibson assembly (NEB) to create pML1335-P_{smyc}Rv2498c. Competent Mtb:*rv2498c*KO were electroporated with pBS-Int (for integrase) with pML1335-P_{smyc}Rv2498c, and then transferred to 5 mL 7H9 medium with supplement (ADC) and Tyloxapol, and left standing overnight at 37 °C. The electroporated cells were plated on 7H11 medium with supplement (OADC) containing 100 mg/mL Hygromycin B to select for complemented colonies. Complementation was checked by PCR (same primers used in gene amplification noted above) with genomic DNA obtained by InstaGene Matrix (Bio-Rad).

Other techniques

DNA sequencing was performed by GATC Biotech (Konstanz, Germany). Protein concentration was determined by bicinchoninic acid (BCA) assay (Pierce BCA Protein Assay Kit), using bovine serum albumin as the standard.

Data analysis

Data were processed by GraphPad PRISM 7.02, Agilent Qualitative Analysis B.07.00, MestReNova 12.0.1-20560, The PyMOL Molecular Graphics System Version 2.2.3.

Crystallization Conditions

Preparations of Rv2498c containing various ligands crystallized under two general conditions, acetate pH 7 and sulphate (or phosphate) pH ~5, yielding different crystal forms (R32 or C2, respectively). Crystals were grown using the sitting drop vapour diffusion method at room temperature. The initial protein solution contained Rv2498c at a concentration of 15 mg/mL in 50 mM HEPES pH 7.4 and 300 mM NaCl.

Prior to data collection, all crystals were transferred to cryoprotectant solutions composed of their mother liquors supplemented with 20% glycerol and flash-cooled in a nitrogen stream at 100K. X-ray diffraction data were collected by LRL-CAT staff at APS beamline 31-ID-D (Advanced Photon Source, Argonne National Laboratory, Argonne, IL) on a Rayonix 225-HE detector (Rayonix). Diffraction intensities were integrated, scaled and merged with the programs DENZO and SCALEPACK.(21) Data collection statistics are given in **Supplementary Table 2**.

For final crystallization conditions for the six complexes

(1) Rv2498c·Mg^{2+}·CoA·Pyruvate: to a protein solution containing 10 mM $MgCl_2$, 40mM pyruvate, and 40 mM acetyl-CoA was added precipitant containing 0.4 M ammonium phosphate pH 4.2. Crystals appeared in one week and exhibited diffraction consistent with the space group C2 with three molecules of the complex per asymmetric unit.

(2) Rv2498c·Mg^{2+}·Pyruvate·Citramalyl-CoA: to a protein solution containing 20 mM $MgCl_2$, 100 mM pyruvate, and 20 mM acetyl-CoA was added a precipitant containing 0.4 M ammonium phosphate pH 4.2. For this sample crystal appeared in three weeks and exhibited diffraction consistent with the space group C2 with tree molecules of the complex per asymmetric unit.

(3) Rv2498c·Mg^{2+}·Acetate: to a protein solution containing 10 mM $MgCl_2$ was added a precipitant containing 1.0 M sodium acetate pH 7.0. Crystals appeared in 4-5 days and exhibited diffraction consistent with the space group R32, with one molecule of the complex per asymmetric unit.

(4) Rv2498c·Mg^{2+}·Acetoacetate: to a protein solution containing 10 mM $MgCl_2$ and 2 M acetoacetate was added a precipitant containing 1.0 M sodium acetate pH 7.0. Crystals appeared in 3 days and exhibited a diffraction pattern consistent with space group R32, with 1 molecule of the complex per asymmetric unit.

(5) Rv2498c·Mg^{2+}·Pyruvate: to a protein solution containing 10 mM $MgCl_2$ and 2 M pyruvate was added a precipitant contained 1.0 M sodium acetate pH 7.0. Crystals appeared in 2 days and exhibited a diffraction pattern consistent with space group R32 with one molecule of the complex per asymmetric unit.

(6) Rv2498c·Mg^{2+}·CoA·Acetoacetate: to a protein solution containing 10 mM $MgCl_2$, 40 mM acetoacetate, and 40 mM Acetyl-CoA was added a precipitant contained 2 M ammonium sulfate pH 5.5. Crystals appeared in one week and exhibited diffraction consistent with the space group C2 with 3 molecules of the complex per asymmetric unit.

Crystallographic Structure Determination and Refinement

Non-CoA ligands: The structures of three of the complexes, Rv2498c·Mg^{2+}·Acetate, Rv2498c·Mg^{2+}·Acetoacetate, and Rv2498c·Mg^{2+}·Pyruvate, were solved by molecular replacement using PHENIX with the apo structure of Rv2498c from *M. tuberculosis* (PDB ID 1U5H; residues 1-221) as a search model.(22, 23)

Automatic model building with ARP/wARP was followed by iterative cycles of manual model building and refinement, performed with COOT and PHENIX, respectively.(23-25) Refinement of the complexes converged at the following values: Rv2498c·Mg^{2+}·Acetate: R_{work}=0.206, R_{free}=0.209 at 1.61Å resolution (PDB entry 6CHU); Rv2498c·Mg^{2+}·Acetoacetate: R_{work}=0.184, R_{free}=0.202 at 1.73Å resolution (PDB entry 6CJ4); Rv2498·Mg^{2+}·Pyruvate: R_{work}=0.195, R_{free}=0.219 at 1.73Å resolution (PDB entry 6CJ3). Refinement statistics are given in Table S2.

The final models of 6CHU, 6CJ3 and 6CJ4 consist of the following:

6CHU – amino acids 1-221 and 250-265 of Rv2498c and one His of the N-terminal affinity tag. All other cloning artifacts along with amino acids 222-249 and 266 to 273 were not observed in the electron density, presumably due to disorder. One magnesium ion and four acetate molecules were also well ordered in the structure and were included in the final model.

6CJ4 – amino acids 1-224 and 251-265 of Rv2498c. All cloning artifacts along with amino acids 225-250 and 266 to 273 were not observed in the electron density, presumably due to

disorder. One magnesium ion, one acetate, and an acetoacetate molecule were also well ordered in the structure and were included in the final model.

6CJ3 – amino acids 2-224 and 250-265 of Rv2498c. All cloning artifacts along with amino acids 1, 225-249 and 266 to 273 were not observed in the electron density, presumably due to disorder. One magnesium ion, three acetates, and two pyruvate molecules were also well ordered in the structure and were included in the final model.

Rv2498c·Mg^{2+}·CoA·Acetoacetate: The complex structure of Rv2498c with Mg^{2+}, acetoacetate and CoA was determined by molecular replacement with PHENIX using the complex of Rv2498c with Mg^{2+} and acetoacetate, determined earlier, as a search model.(22, 23) Several rounds of automated and manual building and refinement, with COOT, ARP/wARP, and PHENIX, respectively, converged with R_{work}=0.217 and R_{free}=0.277 at 2.04Å resolution.(23-25) The final model consists of Rv2498c residues 3-269 in all three chains of the trimeric asymmetric unit (the N-terminal cloning artefacts, affinity tag, and residues 1, 2, and 270-273 were not observed). A magnesium ion, coordinated acetoacetate, and CoA molecule were well ordered in the active sites of three of the Rv2498c subunits and were included in the refined model. Additionally, two glycerol molecules and three sulphate ions were located and included in the final model. The coordinates and structure factors have been deposited in the PDB as entry 6AS5.

Rv2498c·Mg^{2+}·Citramalyl-CoA·Pyruvate: The complex structure of Rv2498c with Mg^{2+}, pyruvate, and citramalyl-CoA was determined by molecular replacement with PHENIX using the complex of Rv2498c with Mg^{2+} and pyruvate, determined earlier, as a search model.(22, 23) Several rounds of manual building and refinement, with COOT and PHENIX, respectively, converged with R_{work}=0.191 and R_{free}=0.233 at 1.83Å resolution.(23-25) The final model consists of Rv2498c residues in three chains of the trimeric asymmetric unit (chain A, 2-268; chain B, 1-268; chain C, 1-267; the N-terminal cloning artefacts, affinity tag, and residues 1 in chain A, 269-273 in chains A and B, and 268-273 in chain C were not observed). A magnesium ion was located in each active site; two citramalyl-CoA molecules (presumably arising as the product of enzyme-mediated reaction between pyruvate and acetyl-CoA both of which were present in the crystallization milieu) were well ordered in the active sites of chains A and B and were included in the model. The magnesium ion in the active site of chain C was coordinated by a pyruvate molecule. Additionally, two glycerol molecules and three phosphate ions and five chlorides were located and included in the final model. The coordinates and structure factors have been deposited in the PDB as entry PDB entry 6AQ4.

Rv2498c·Mg^{2+}·CoA·Pyruvate: The complex structure of Rv2498c with Mg^{2+}, pyruvate and CoA was solved by molecular replacement with PHENIX using the complex of Rv2498c with Mg^{2+} and pyruvate, determined earlier, as a search model.(22, 23) Several rounds of building and refinement, with COOT and PHENIX, respectively, converged with R_{work}=0.219 and R_{free}=0.262 at 1.72Å resolution.(23-25) The final model consists of Rv2498c residues 2-269 in each of three chains of the trimeric asymmetric unit (the N-terminal cloning artifacts, affinity tag, and residues 1 and 270-273 were not observed). A magnesium ion, coordinated pyruvate, and CoA molecule were observed in the active sites of three of the Rv2498c subunits and were included in the refined model. Additionally, five glycerol molecules and three phosphate ions were located and included in the final model. The coordinates and structure factors have been deposited in the PDB as entry 6ARB.

Rv2498c·Mg^2+^·CoA·Acetoacetate: The complex structure of Rv2498c with Mg^{2+}, acetoacetate and CoA was solved by molecular replacement with PHENIX using the complex of Rv2498c with Mg^{2+} and acetoacetate, determined earlier, as a search model.(22, 23) Several rounds of automated and manual building and refinement, with COOT, ARP/wARP, and PHENIX, respectively, converged with R_{work}=0.217 and R_{free}=0.277 at 2.04Å resolution.(23-25) The final model consists of Rv2498c residues 3-269 in all three chains of the trimeric asymmetric unit (the N-terminal cloning artifacts, affinity tag, and residues 1, 2, and 270-273 were not observed). A magnesium ion, coordinated acetoacetate, and CoA molecule were well ordered in the active sites of three of the Rv2498c subunits and were included in the refined model. Additionally, two glycerol molecules and three sulfate ions were located and included in the final model. The coordinates and structure factors have been deposited in the PDB as entry 6AS5.

Rv2498c·Mg^{2+}·Citramalyl-CoA·Pyruvate: The complex structure of Rv2498c with Mg^{2+}, pyruvate, and citramalyl-CoA was solved by molecular replacement with PHENIX using the complex of Rv2498c with Mg^{2+} and pyruvate, determined earlier, as a search model.(22, 23) Several rounds of manual building and refinement, with COOT and PHENIX, respectively, converged with R_{work}=0.191 and R_{free}=0.233 at 1.83Å resolution.(23-25) The final model consists of Rv2498c residues in three chains of the trimeric asymmetric unit (chain A, 2-268; chain B, 1-268; chain C, 1-267; the N-terminal cloning artifacts, affinity tag, and residues 1 in chain A, 269-273 in chains A and B, and 268-273 in chain C were not observed). A magnesium ion was located in each active site; two citramalyl-CoA molecules (presumably arising as the product of enzyme-mediated reaction between pyruvate and acetyl-CoA both of which were present in the crystallization milieu) were well ordered in the active sites of chains A and B and were included in the model. The magnesium ion in the active site of chain C was coordinated by a pyruvate molecule. Additionally, two glycerol molecules and three phosphate ions and five chlorides were located and included in the final model. The coordinates and structure factors have been deposited in the PDB as entry PDB entry 6AQ4.

Final crystallographic details for all Rv2498c X-ray structures are provided in **Supplementary Table 2**.

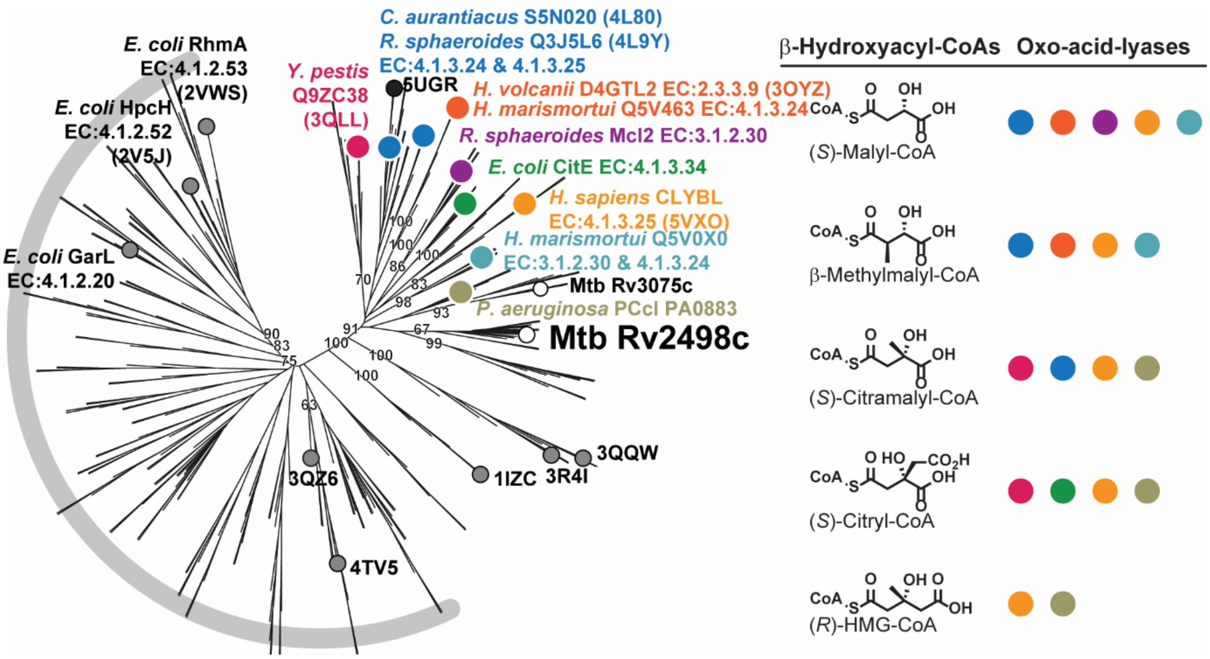

Figure S1. Phylogenetic tree of Rv2498c and related lyases pinpoints widespread substrate multiplicity. Neighbor-joining protein phylogenetic tree of the PFAM HpcH/HpaI aldolase/citrate lyase family (PF03328). The tree is mainly divided in two lobes: the aldehyde-lyases (EC: 4.1.2) in grey and the oxo-acid-lyases (EC: 4.1.3). Most of the clusters contain only bacterial sequences, but archaeal- and fungal-only groups are also present. There is only one cluster that includes metazoan sequences, that of the itaconate-detoxifying CLYBL.(26) The irregular taxonomic distribution of the bacterial branches suggests an extensive lateral transfer. The only two enzymes in the family in the Mtb genome are highlighted with white circles. Colored circles denote experimentally characterized oxo-acid lyases shown with their respective β-hydroxyacyl-CoA substrates; PDB, UnitProtK and/or E.C. codes are provided. The Rv2498c cluster is actinobacterial-specific and contains only uncharacterized sequences (seventy-two non-redundant UnitProt entries). Other closely related groups are the *bona fide* CitE (*e.g.* P0A9I1), the archaeal L-malyl-CoA/β-methylmalyl-CoA lyases involved in the methylaspartate cycle (*e.g.* Q5V463), and the (*S*)-citramalyl-CoA lyases from *Y. pestis* (Q9ZC38) and *P. aeruginosa* (Pa0883; Q9I562).(27, 28) Characterized enzymes include the *Y. pestis* (*S*)-citramalyl-CoA lyase involved in itaconate dissimilation; *R. sphaeroides* (Mcl1) and *C. aurantiacus* (Mcl) L-malyl-CoA/β-methylmalyl-CoA lyases involved in CO_2 fixation and acetate assimilation; archaeal *Haloferax volcanii* AceB and *Haloarcula marismortui* CitE1 L-malyl-CoA/β-methylmalyl-CoA lyases, involved in the methylaspartate cycle; *R. sphaeroides* (Mcl2) L-malyl-CoA thioesterase; *E. coli* CitE (*e.g.* P0A9I1) involved in TCA anaplerosis; human CLYBL (*S*)-citramalyl-CoA lyase involve in itaconate dissimilation; archaeal *H. marismortui* AceB L-malyl-CoA/β-methylmalyl-CoA lyase/thioesterase; and *P. aeruginosa* (*S*)-citramalyl-CoA lyase involved in itaconate dissimilation.

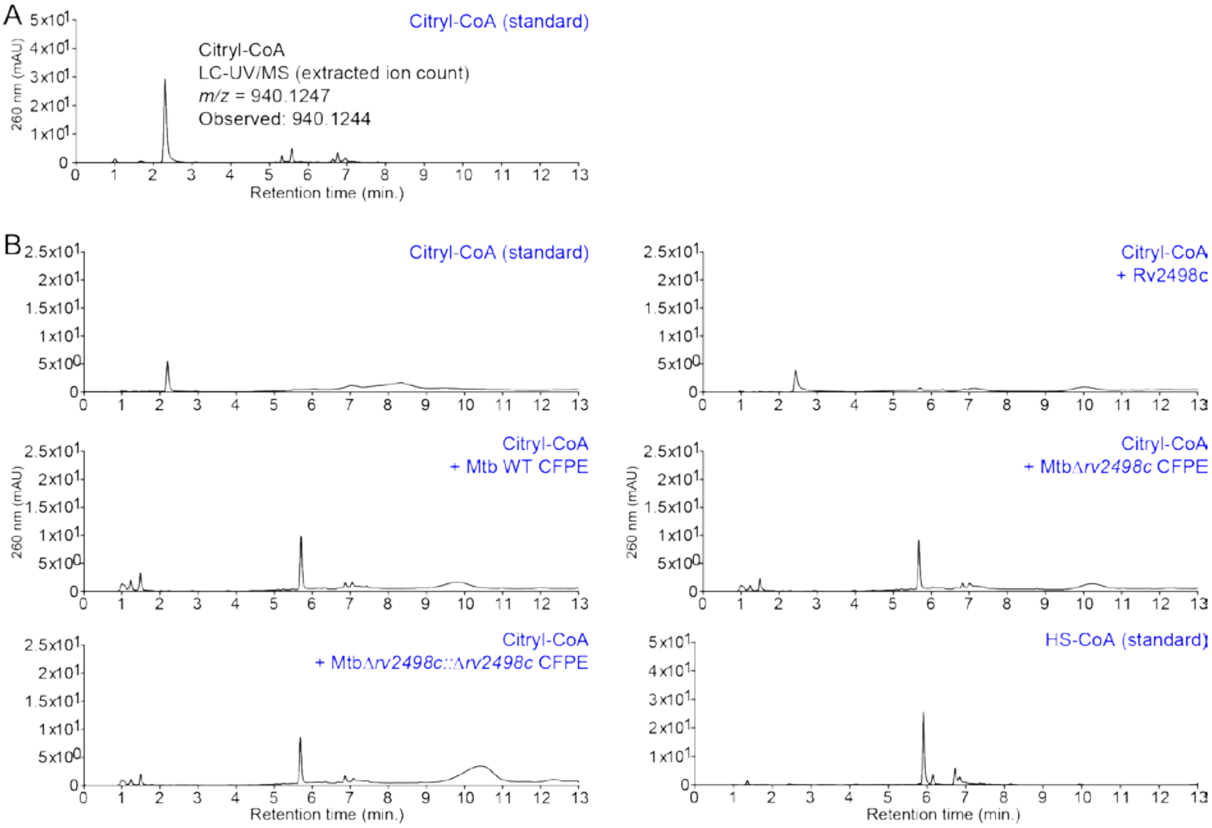

Figure S2. (*S*)-Citryl-CoA is not a substrate for Rv2498c. (**A**) LC-UV/MS for (*S*)-Citryl-CoA standard (2.3 min), with a calculated m/z 940.1247 and observed 940.1244. (**B**) HPLC chromatograms of (*S*)-Citryl-CoA standard with Rv2498c, CFPEs (Mtb WT, Rv2498c, and complement), and HS-CoA standard (5.9 min) The addition of Rv2498c to (*S*)-Citryl-CoA did not result in product formation, suggesting (*S*)-Citryl-CoA is not a substrate for Rv2498c.

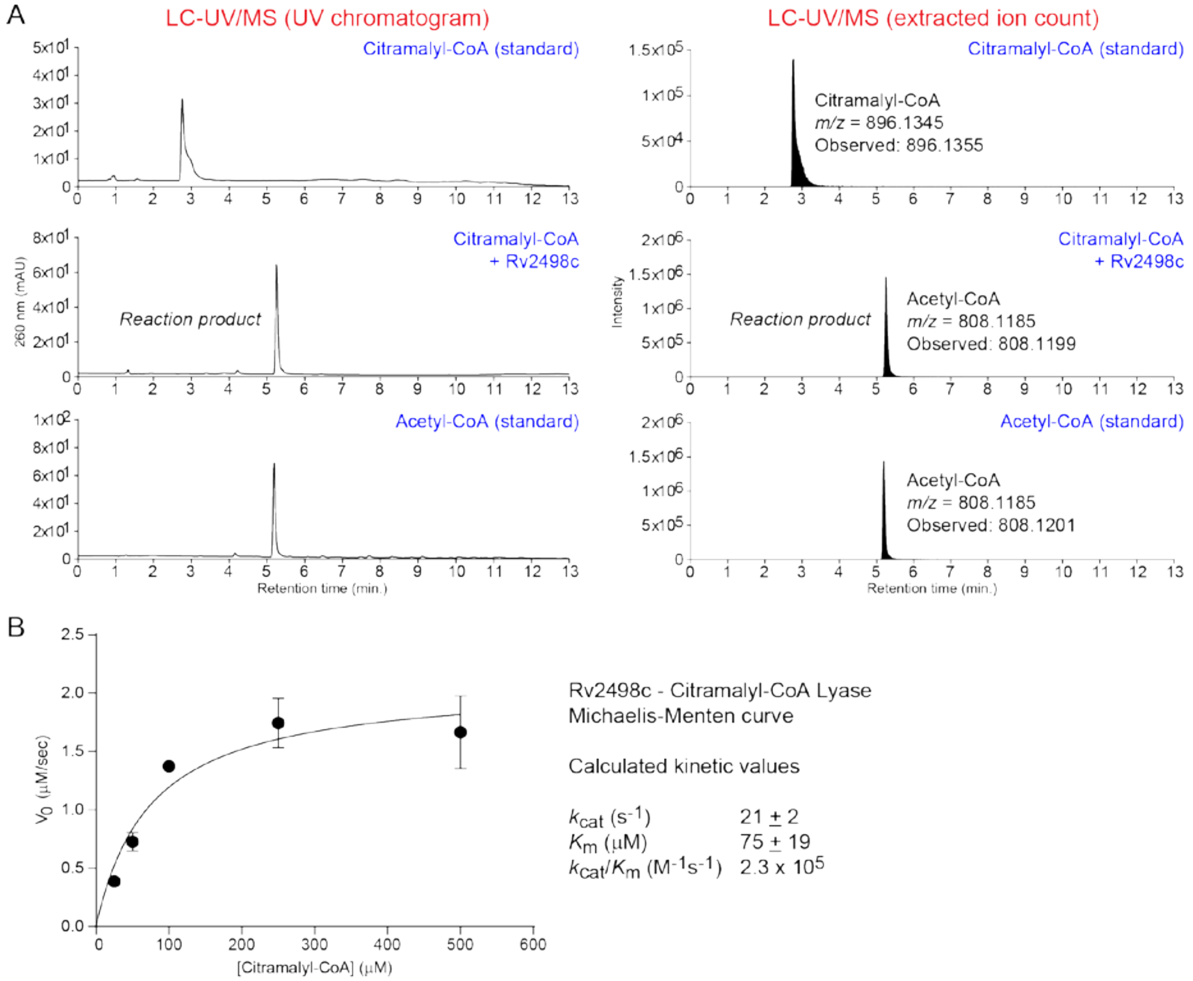

Figure S3. LC-UV/MS of (*S*)-Citramalyl-CoA with Rv2498c. (**A**) UV-Vis (left) and extracted- ion (right) chromatograms of substrate (*S*)-citramalyl-CoA standard (2.8 min), reaction mixture containing (*S*)-citramalyl-CoA with 0.5 μM Rv2498c, and product Acetyl-CoA standard (5.2 min). (**B**) Michaelis-Menten curve of the carbon-carbon cleavage of (*S*)-citramalyl-CoA by Rv2498c.

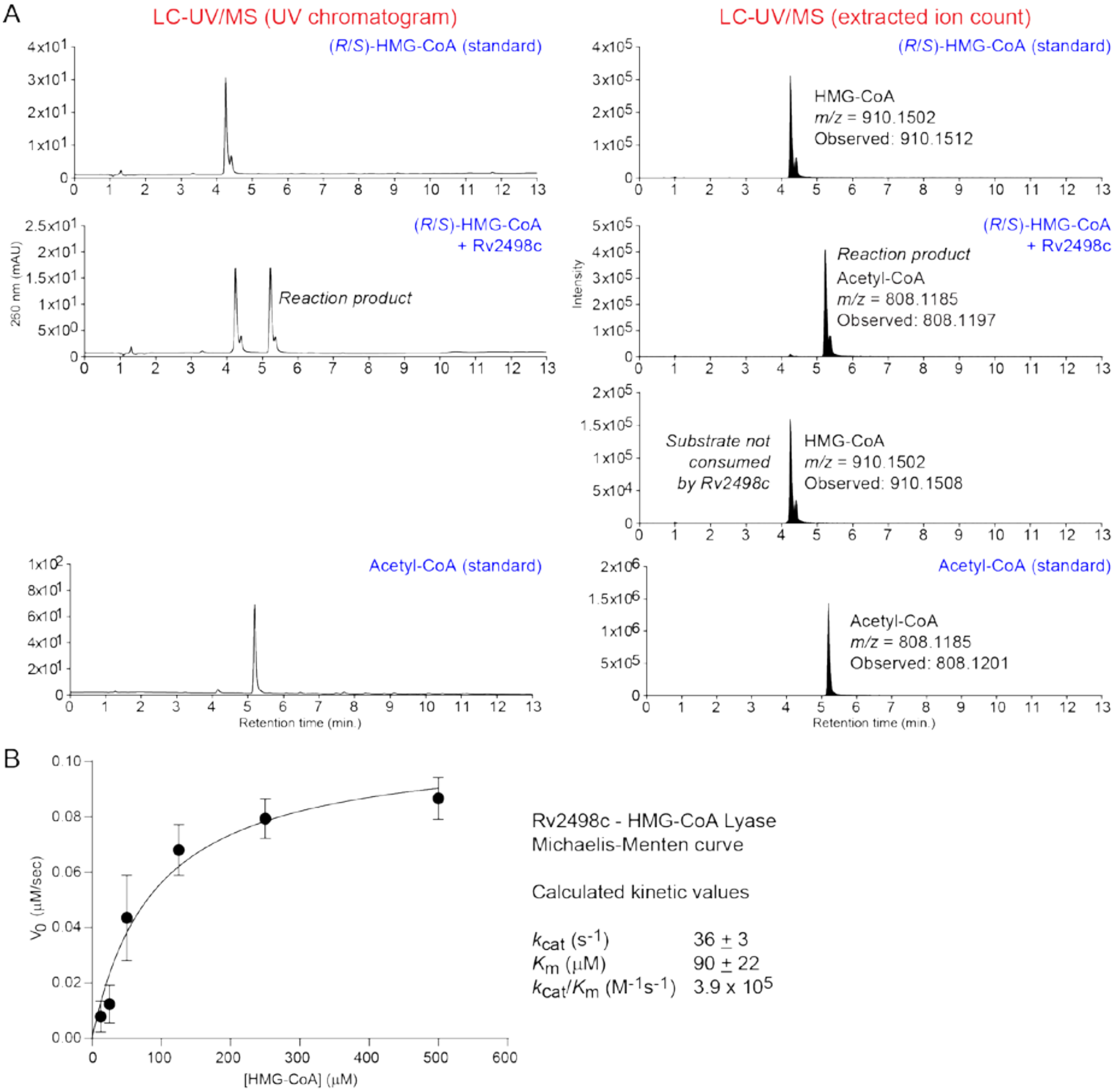

Figure S4. LC-UV/MS of (*R*/*S*)-HMG-CoA-CoA with Rv2498c. (**A**) UV-Vis (left) and extracted- ion (right) chromatograms of substrate (*R*/*S*)-HMG-CoA standard (4.2 min), reaction mixture containing (*R*/*S*)-citramamyl-CoA with 0.5 µM Rv2498c, and product Acetyl-CoA standard (5.2 min). (**B**) Michaelis-Menten curve of the carbon-carbon cleavage of (*R*)-HMG-CoA by Rv2498c.

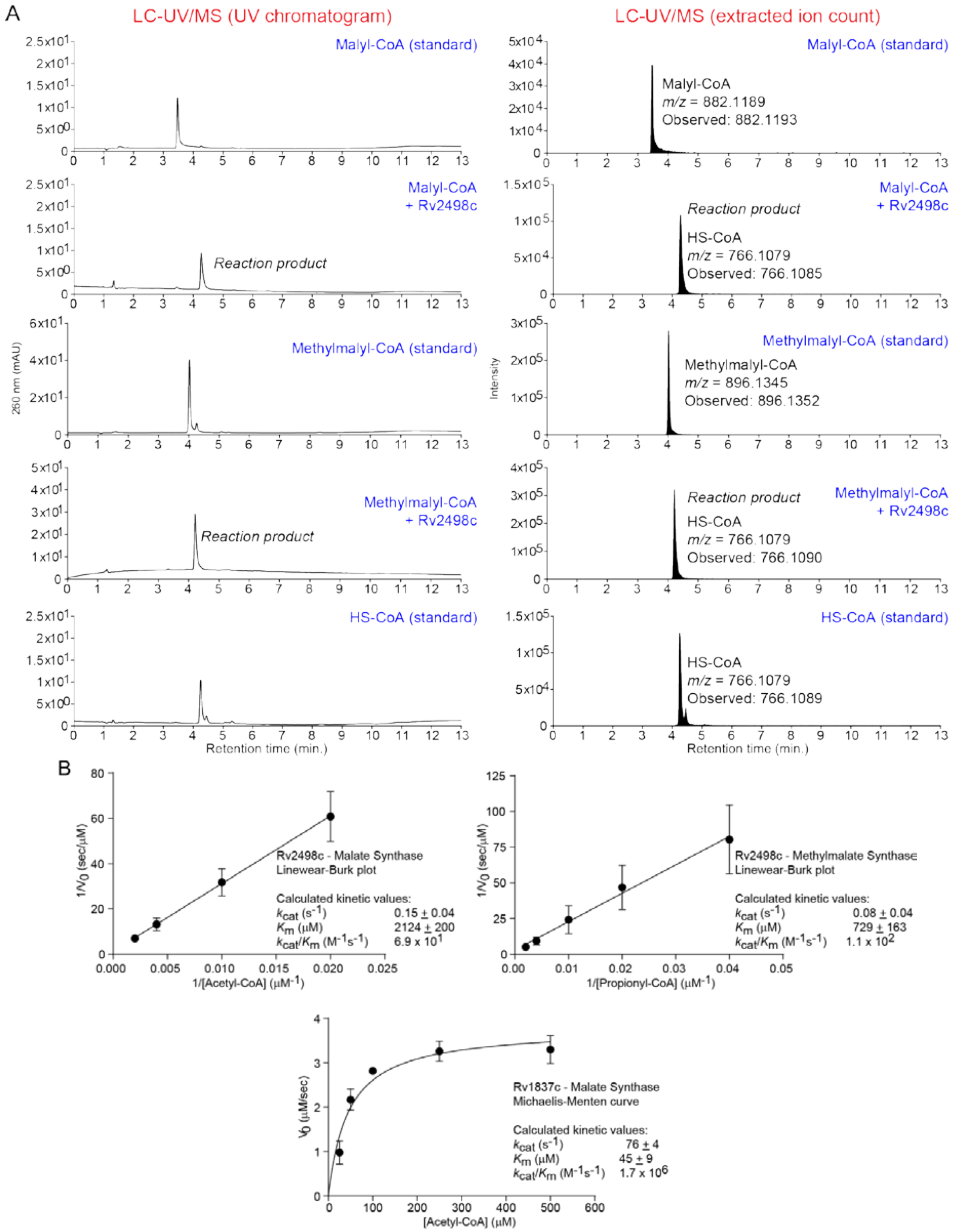

Figure S5. LC-UV/MS of Malyl-CoA and Methylmalyl-CoA with Rv2498c. (**A**) UV-Vis (left) and extracted- ion (right) chromatograms of substrate (*S*)-Malyl-CoA standard (3.5 min), reaction mixture containing (*S*)-Malyl-CoA with 0.5 μM Rv2498c, substrate Methylmalyl-CoA standard (4.0 min), reaction mixture containing Methylmalyl-CoA with 0.5 μM Rv2498c, and product HS-CoA standard (4.3 min). (**B**) Lineweaver-Burk plot of the synthesis of malate (glyoxylate:Acetyl-CoA, 1:200) and methylmalate (glyoxylate:Propionyl-CoA, 1:200) by Rv2498c, and Michaelis-Menten curve of the synthesis of malate (glyoxylate:Acetyl-CoA, 1:2) by the *bona fide* malate synthase, Rv1837c.

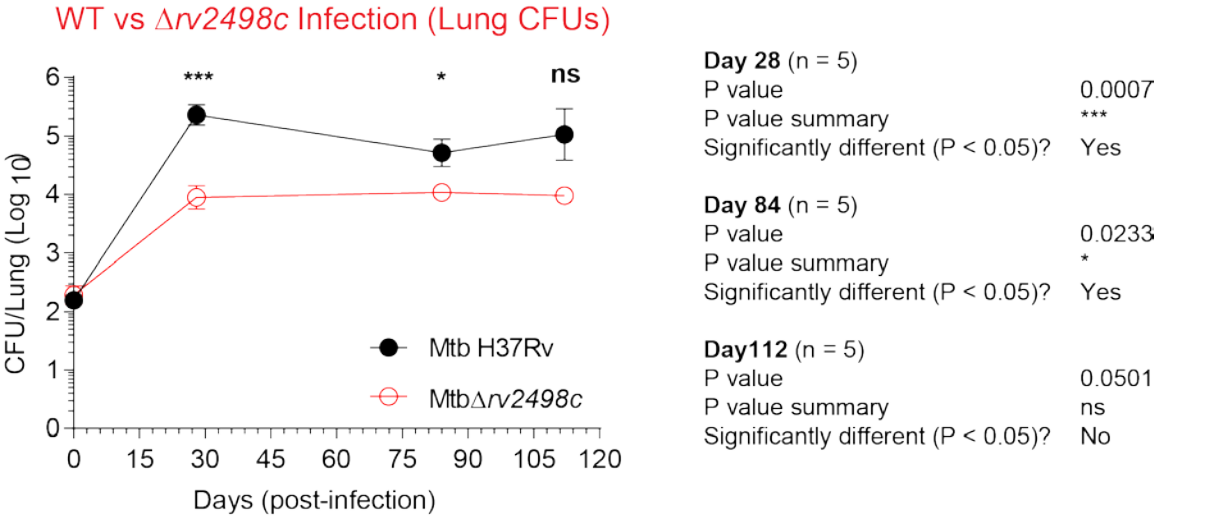

Figure S6. Mouse aerosol infection study with Mtb H37Rv and Δ*rv2498c* mutant. Rv2498cKO displays at least one log reduction in colony forming units (CFUs) in lungs at day 28, 84, 112 post infection, compared to parent strain Mtb H37Rv. Data points are from an *n* of 5 mice per group. The significance was determined by Student's *t*-test with $*P \leq 0.05$, $**P \leq 0.001$, or $***P \leq 0.0001$.

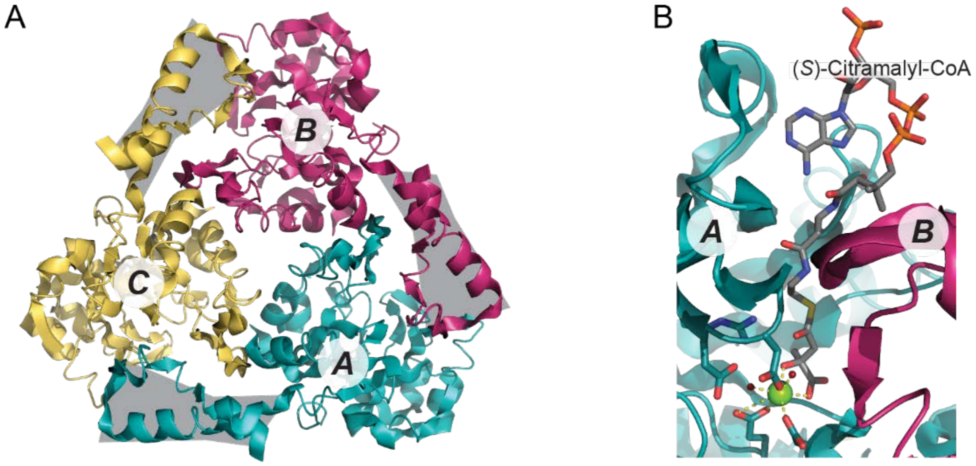

Figure S7. Crystal structures of Rv2498c with C-terminal domain and (*S*)-Citramamyl-CoA. (**A**) Ribbon representation of trimeric Rv2498c with protomers A, B, and C (PDB: 6CJ4). The structured C-terminal domain is highlighted in grey. (**B**) Ribbon and stick representation of Rv2498c bound to (*S*)-citramalyl-CoA (PDB: 6AQ4), highlighting the contribution of the C-terminal domain of protomer B (magenta) to the binding pocket of protomer A (cyan).

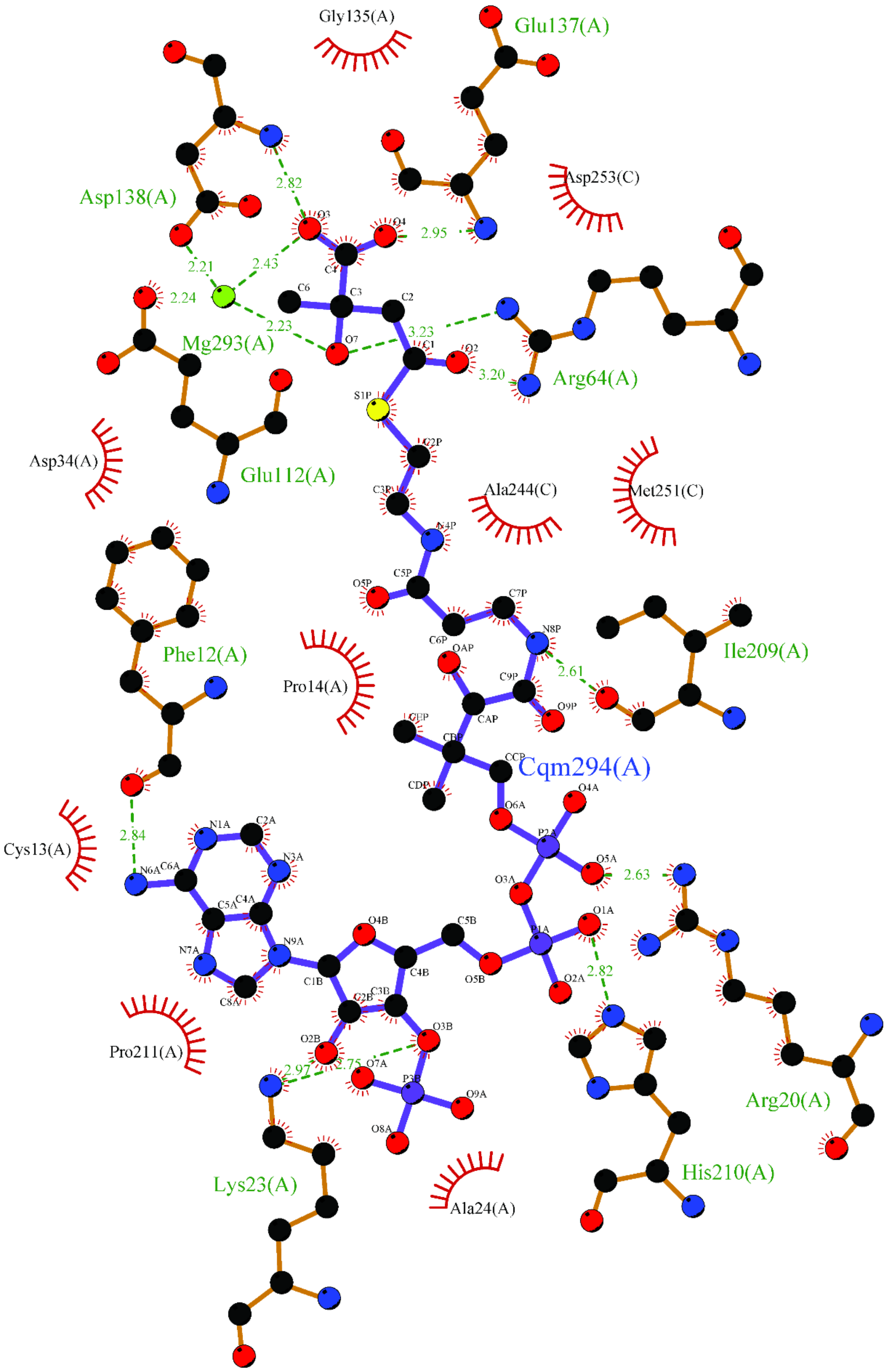

Figure S8. Stick representation of Rv2498c-ligand complex. The Rv2498c-ligand complex of active site residues responsible for interaction with magnesium metal and (*S*)-citramalyl-CoA. The image was generated with Ligplot+.

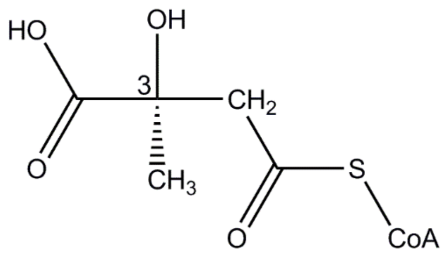

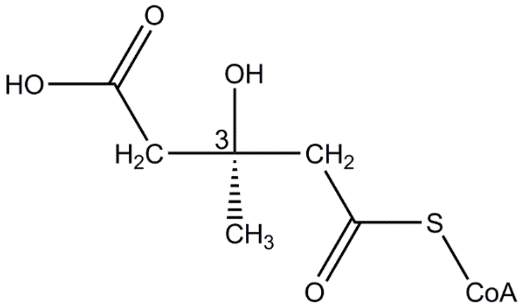

(*S*)-citramalyl-CoA (*R*)-HMG-CoA

Figure S9. Stereochemistry of 3-carbon position of β-hydroxyl-acyl-CoA thioesters. Stereochemistry at the 3 carbon positions of *S*-citramalyl-CoA (left) and *R*-HMG-CoA (right).

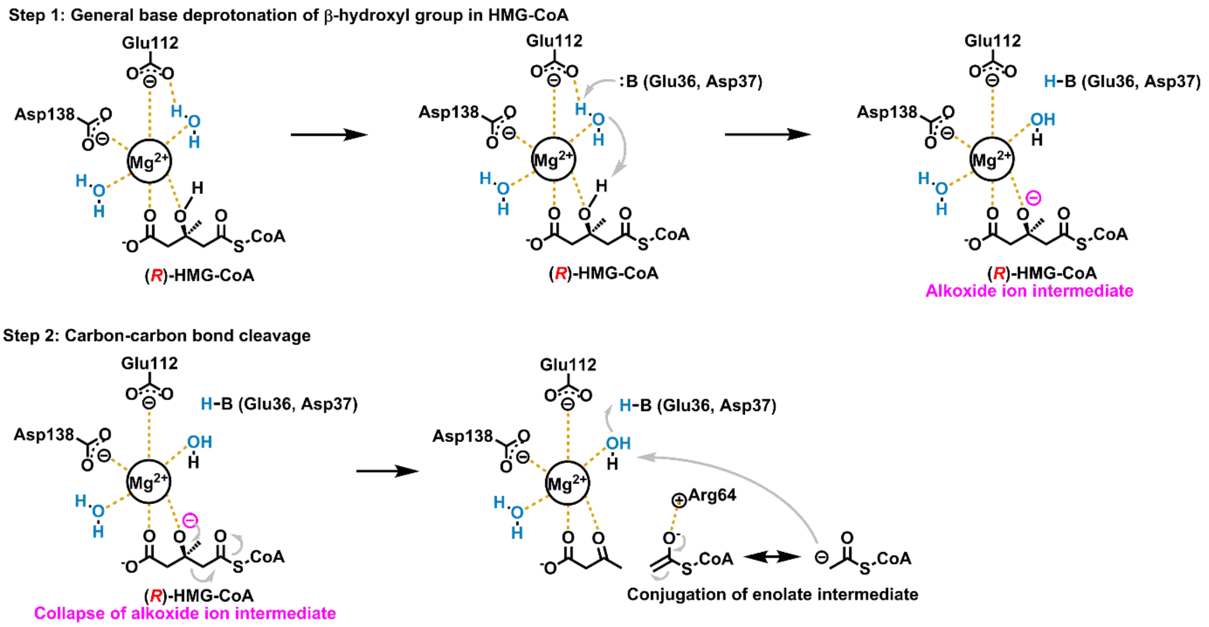

Figure S10. A proposed mechanism for Rv2498c C-C cleavage of β-hydroxyl-acyl-CoA thioesters. The first step in the reaction is the base catalysed deprotonation of the β-hydroxyl group of HMG-CoA *via* metal-bound water-mediated proton abstraction. Based on our structures, the general base is either Glu36 or Asp37, both at close proximity (less than 3 angstroms) to the water molecules. This first step generates an alkoxide intermediate. The second step is the C-C bond cleavage as a result of the collapse of the alkoxide resulting in the two reaction products: acetoacetate and acetyl-CoA. The Arg64 is suggested to partially involve in the enolisation of the acetyl-CoA intermediate. The general base is likely regenerated by the metal-bound water-mediated proton abstraction.

Table S1. CoA-thioesters.

	**Substrate**
1	Coenzyme A sodium salt hydrate
2	HMG-Coenzyme A sodium salt hydrate
3	Acetyl Coenzyme A lithium salt
4	*n*-Heptadecanoyl CoA lithium salt
5	Acetoacetyl coenzyme A sodium salt
6	Isobutyryl coenzyme A lithium salt
7	Hexanoyl coenzyme A trilithium salt
8	Octanoyl coenzyme A lithium salt
9	Palmitoyl coenzyme A potassium salt
10	Palmitoleoyl coenzyme A lithium salt
11	Malonyl coenzyme A lithium salt
12	Succinyl coenzyme A sodium salt
13	Glutaryl coenzyme A lithium salt
14	Phenylacetyl coenzyme A lithium salt
15	β-hydroxylbutryl coenzyme A lithium salt
16	Methylmalonyl coenzyme A tetralithium salt
17	Myristoyl coenzyme A lithium salt
18	Crotonyl coenzyme A trilithium salt
19	Isovaleryl coenzyme A lithium salt
20	*n*-Propionyl coenzyme A lithium salt
21	*n*-Butyryl coenzyme A lithium salt
22	β-Methylcrotonyl coenzyme A lithium salt
23	Decanoyl coenzyme A monohydrate
24	Lauroyl coenzyme A lithium salt
25	Stearoyl-coenzyme A lithium salt
26	Oleoyl coenzyme A lithium salt
27	Linoleoyl coenzyme A lithium salt
28	3'dephosphocoenzyme A
29	Benzoyl coenzyme A lithium salt
30	Arachidonyl coenzyme A lithium salt

Table S2. X-ray data collection and refinement statistics for complexes of Rv2498c from *Mycobacterium tuberculosis*.

	Rv2498c·Mg $^{2+}$· Acetate	Rv2498c·Mg^{2+}· Acetoacetate	Rv2498c·Mg^{2+}· Pyruvate	Rv2498c·Mg^{2+}· Acetoacetate·CoA	Rv2498c·Mg^{2+}· Pyruvate· CoA	Rv2498c·Mg^{2+}·Pyruvate· Citramalyl-CoA
**Data collection**						
Space group	R32	R32	R32	C2	C2	C2
No. of mol. in asym. unit	1	1	1	3	3	3
Cell dimensions						
a, *b*, *c* (Å)	91.13,91.13,220.29	91.67,91.67,221.39	91.99,91.99,221.14	126.58,73.00,85.79	127.53,73.49,86.46	138.54,88.36,81.25
β (°)				98.83	99.14	108.54
Resolution (Å)	1.61	1.73	1.73	2.04	1.72	1.83
No. of unique reflections	88184	72324	72884	48935	163623	154090
R_{merge}	0.073	0.059	0.064	0.094	0.084	0.063
$I / \sigma I$	12.2	15.5	17.3	14.6	11.6	10.9
Completeness (%)	99.8	99.8	99.9	99.6	98.9	94.8
**Refinement**						
Resolution (Å)	25.0-1.61	25.0-1.73	25.0-1.73	25.0-2.04	25.0-1.72	25.0-1.83
R_{cryst}	0.206	0.184	0.195	0.217	0.219	0.191
R_{free}	0.209	0.202	0.219	0.277	0.262	0.233
No. atoms						
Protein	1800	1813	1826	5964	5979	6012
Waters	165	134	147	235	445	473
R.m.s deviations						
Bond lengths (Å)	0.007	0.007	0.007	0.007	0.007	0.007
Bond angles (°)	1.0	1.1	1.0	1.1	1.1	1.0
Bound ligand	4ACT	1AAE,1ACT	2PYR,3ACT	3COA,3AAE,2GOL	3COA,3PYR,5GOL	2CQM,1PYR,2GOL
Bound ions	1Mg^{2+}	1Mg^{2+}	1Mg^{2+}	3Mg^{2+},3SO$_4^{2-}$	3 Mg^{2+},3PO$_4^{3-}$	3Mg^{2+} ,3PO$_4^{3-}$, 5Cl^{-1}
PDB entry	6CHU	6CJ4	6CJ3	6AS5	6ARB	6AQ4

References

1. Huff J, Czyz A, Landick R, & Niederweis M (2010) Taking phage integration to the next level as a genetic tool for mycobacteria. *Gene* 468(1-2):8-19.
2. Finn RD*, et al.* (2016) The Pfam protein families database: towards a more sustainable future. *Nucleic Acids Res* 44(D1):D279-285.
3. Pettersen EF*, et al.* (2004) UCSF Chimera--a visualization system for exploratory research and analysis. *J Comput Chem* 25(13):1605-1612.
4. Konagurthu AS, Whisstock JC, Stuckey PJ, & Lesk AM (2006) MUSTANG: a multiple structural alignment algorithm. *Proteins* 64(3):559-574.
5. Katoh K & Standley DM (2013) MAFFT multiple sequence alignment software version 7: improvements in performance and usability. *Mol Biol Evol* 30(4):772-780.
6. Waterhouse AM, Procter JB, Martin DM, Clamp M, & Barton GJ (2009) Jalview Version 2--a multiple sequence alignment editor and analysis workbench. *Bioinformatics* 25(9):1189-1191.
7. Darriba D, Taboada GL, Doallo R, & Posada D (2011) ProtTest 3: fast selection of best-fit models of protein evolution. *Bioinformatics* 27(8):1164-1165.
8. Felsenstein J (1989) PHYLIP - Phylogeny Inference Package (Version 3.2). *Cladistics* 5:164-166.
9. Schmidt HA, Strimmer K, Vingron M, & von Haeseler A (2002) TREE-PUZZLE: maximum likelihood phylogenetic analysis using quartets and parallel computing. *Bioinformatics* 18(3):502-504.
10. Gascuel O (1997) BIONJ: an improved version of the NJ algorithm based on a simple model of sequence data. *Mol Biol Evol* 14(7):685-695.
11. Edgar RC (2004) MUSCLE: a multiple sequence alignment method with reduced time and space complexity. *BMC Bioinformatics* 5:113.
12. Guindon S & Gascuel O (2003) A simple, fast, and accurate algorithm to estimate large phylogenies by maximum likelihood. *Syst Biol* 52(5):696-704.
13. Huson DH*, et al.* (2007) Dendroscope: An interactive viewer for large phylogenetic trees. *BMC Bioinformatics* 8:460.
14. Zarzycki J & Kerfeld CA (2013) The crystal structures of the tri-functional Chloroflexus aurantiacus and bi-functional Rhodobacter sphaeroides malyl-CoA lyases and comparison with CitE-like superfamily enzymes and malate synthases. *BMC Struct Biol* 13:28.
15. Buckel W, Ziegert K, & Eggerer H (1973) Acetyl-Coa-Dependent Cleavage of Citrate on Inactivated Citrate Lyase. *European Journal of Biochemistry* 37(2):295-304.
16. de Carvalho LP*, et al.* (2010) Metabolomics of Mycobacterium tuberculosis reveals compartmentalized co-catabolism of carbon substrates. *Chem Biol* 17(10):1122-1131.
17. Larrouy-Maumus G*, et al.* (2013) Discovery of a glycerol 3-phosphate phosphatase reveals glycerophospholipid polar head recycling in Mycobacterium tuberculosis. *Proc Natl Acad Sci U S A* 110(28):11320-11325.
18. Pesek JJ, Matyska MT, Fischer SM, & Sana TR (2008) Analysis of hydrophilic metabolites by high-performance liquid chromatography-mass spectrometry using a silica hydride-based stationary phase. *J Chromatogr A* 1204(1):48-55.
19. Lange M & Malyusz M (1994) Fast method for the simultaneous determination of 2-oxo acids in biological fluids by high-performance liquid chromatography. *J Chromatogr B Biomed Appl* 662(1):97-102.
20. Parish T & Stoker NG (2000) Use of a flexible cassette method to generate a double unmarked Mycobacterium tuberculosis tlyA plcABC mutant by gene replacement. *Microbiology* 146 (Pt 8):1969-1975.
21. Otwinowski Z & Minor W (1997) Processing of X-ray diffraction data collected in oscillation mode. *Methods Enzymol* 276:307-326.

22. Goulding CW, *et al.* (2007) The structure and computational analysis of Mycobacterium tuberculosis protein CitE suggest a novel enzymatic function. *J Mol Biol* 365(2):275-283.
23. Adams PD, *et al.* (2010) PHENIX: a comprehensive Python-based system for macromolecular structure solution. *Acta Crystallogr D Biol Crystallogr* 66(Pt 2):213-221.
24. Emsley P & Cowtan K (2004) Coot: model-building tools for molecular graphics. *Acta Crystallogr D Biol Crystallogr* 60(Pt 12 Pt 1):2126-2132.
25. Langer G, Cohen SX, Lamzin VS, & Perrakis A (2008) Automated macromolecular model building for X-ray crystallography using ARP/wARP version 7. *Nat Protoc* 3(7):1171-1179.
26. Shen H, *et al.* (2017) The Human Knockout Gene CLYBL Connects Itaconate to Vitamin B12. *Cell* 171(4):771-782 e711.
27. Khomyakova M, Bukmez O, Thomas LK, Erb TJ, & Berg IA (2011) A methylaspartate cycle in haloarchaea. *Science* 331(6015):334-337.
28. Sasikaran J, Ziemski M, Zadora PK, Fleig A, & Berg IA (2014) Bacterial itaconate degradation promotes pathogenicity. *Nat Chem Biol* 10(5):371-377.
